# Optogenetic modulation of hippocampal oscillations ameliorates spatial cognition and hippocampal dysrhythmia following early-life seizures

**DOI:** 10.1016/j.nbd.2023.106021

**Published:** 2023-01-28

**Authors:** Francisco Velasquez, Conor Dickson, Michelle L. Kloc, Carmel A. Schneur, Jeremy M. Barry, Gregory L. Holmes

**Affiliations:** Epilepsy Development and Cognition Group, Department of Neurological Sciences, University of Vermont, Larner College of Medicine, Burlington, VT, USA

**Keywords:** Epilepsy, Seizures, Cognition, Optogenetics, Theta, Dysrhythmia

## Abstract

There is increasing human and animal evidence that brain oscillations play a critical role in the development of spatial cognition. In rat pups, disruption of hippocampal rhythms via optogenetic stimulation during the critical period for memory development impairs spatial cognition. Early-life seizures are associated with long-term deficits in spatial cognition and aberrant hippocampal oscillatory activity. Here we asked whether modulation of hippocampal rhythms following early-life seizures can reverse or improve hippocampal connectivity and spatial cognition. We used optogenetic stimulation of the medial septum to induce physiological 7 Hz theta oscillations in the hippocampus during the critical period of spatial cognition following early-life seizures.

Optogenetic stimulation of the medial septum in control and rats subjected to early-life seizures resulted in precisely regulated frequency-matched hippocampal oscillations. Rat pups receiving active blue light stimulation performed better than the rats receiving inert yellow light in a test of spatial cognition. The improvement in spatial cognition in these rats was associated with a faster theta frequency and higher theta power, coherence and phase locking value in the hippocampus than rats with early-life seizures receiving inert yellow light. These findings indicate that following early life seizures, modification of hippocampal rhythms may be a potential novel therapeutic modality.

## 1. Introduction

Encoding and retrieval of information regarding one’s environment and spatial orientation are major components of spatial memory. Organized expression of hippocampal network oscillations plays a central role in the generation and coordination of memory (Ego-Stengel and Wilson, 2010; Montgomery and Buzsaki, 2007; Nyhus and Curran, 2010; Winson, 1978; Gao et al., 2018). The ontogeny of spatial memory in rodents, which is considered equivalent to declarative memory in humans (Bunsey and Eichenbaum, 1996; Crystal and Smith, 2014; Eichenbaum and Cohen, 2014), is precisely orchestrated with stereotypical patterns of electrical signaling propagating through developing neural circuits to establish connections that optimize information processing (Stryker and Harris, 1986; Kirkby et al., 2013).

Hippocampal oscillatory activity emerges and develops within the first three postnatal weeks in rodents (Lahtinen et al., 2002; Leblanc and Bland, 1979; Leinekugel et al., 2002; Langston et al., 2010). This patterned oscillatory activity in the medial entorhinal cortex-hippocampal circuit finely tunes the firing rates of emerging grid and place cells (Martin and Berthoz, 2002; McNaughton et al., 2006; Couey et al., 2013; Moser et al., 2014; Kropff and Treves, 2008). The maturing of oscillatory activity in the hippocampus parallels allocentric spatial learning and memory which arises approximately between postnatal (P) day 21–25 in rodents (Ainge and Langston, 2012; Akers et al., 2012; Albani et al., 2014; Guskjolen et al., 2017; Rudy et al., 1987; Tan et al., 2017; Baram et al., 2019; Wills et al., 2014). The P21–25 age range is therefore considered a critical period for processing memories which depends on activity and plasticity mechanisms within the developing hippocampus (Gao et al., 2018; Travaglia et al., 2016a; Travaglia et al., 2016b).

Whereas normal activity patterns are required for circuit maturation, aberrant neuronal activity can disrupt spatial cognition. This is particularly important in the case of massive bursts of synchronized network activity which occur during seizures. Mounting clinical evidence (Berg and Rychlik, 2015; Berg et al., 2012; Vasconcellos et al., 2001; Berg et al., 2004) points to a deleterious impact of seizures on cognition, particularly when they occur in the context of a developing brain. Children with pharmacoresistant epilepsy developing before or during the critical period are at very high risk for cognitive impairment. Likewise, in animal models, recurrent (Liu et al., 1999; Karnam et al., 2009a; Holmes et al., 2015) or prolonged (Dube et al., 2009; Barry et al., 2015; Patterson et al., 2017) seizures before or during the critical period have long-standing effects on spatial cognition, hippocampal rhythms and place cell firing patterns. A recent study suggested that rather than the seizures, the dysrhythmia of normal hippocampal rhythms occurring during the critical period may be the major factor in causing cognitive impairment (Kloc et al., 2020).

While dysrhythmia before or during the critical period of spatial cognition can result in longstanding cognitive impairment, whether such deficits can be reversed or ameliorated through modulation of hippocampal rhythms is not known. Here we asked whether deficits in spatial cognition and hippocampal connectivity following early-life seizures could be modified by physiological theta frequency stimulation during the critical period of spatial memory development.

## 2. Methods

All procedures were approved by the University of Vermont’s Institutional Animal Care and Use Committee and conducted in accordance with guidelines from the National Institutes of Health. The results of this study are based on 42 male Sprague-Dawley (Charles River, Montreal) rats. At the time this study was initially designed, both male and female rats were to be included. Following our observation that ELS did not lead to spatial deficits, as assessed in the active avoidance task in female rats (Niedecker et al., 2021), we used only male pups.

Pups were weaned at P21 and group housed until approximately P35, at which time they were individually housed. Rats were maintained on a 12-h light/dark cycle. Experimental design, performance and analysis aimed to adopt guidelines for rigor and reproducibility in science (Landis et al., 2012). Group sizes were determined a priori, and animals were randomly assigned to experimental groups. In the behavioral studies, experimenters were not blinded to treatment group.

### Experimental Overview:

**Fig. 1.**
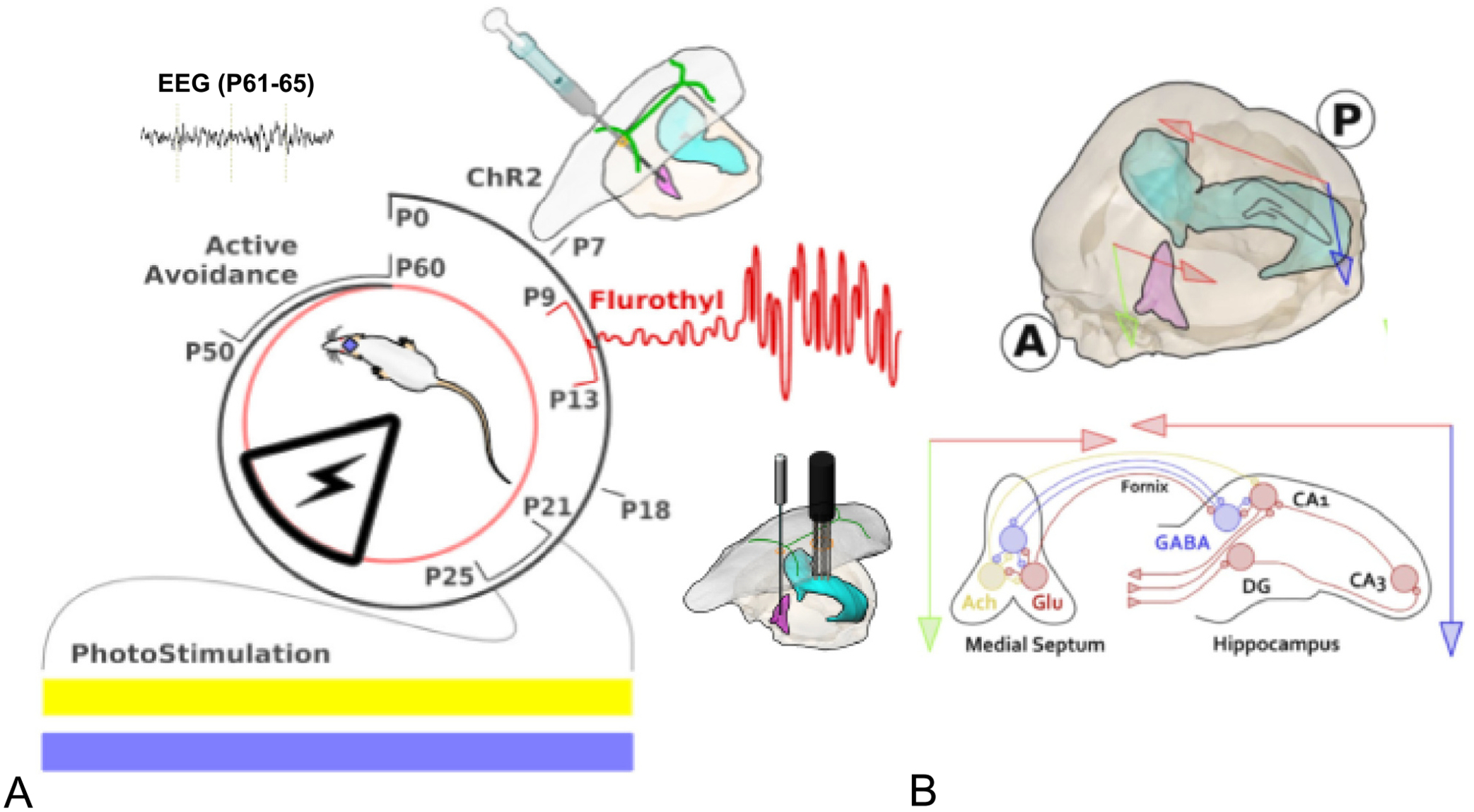
Graphical representation of the timeline of experiment and anatomy of the medial septum-hippocampal spatial circuit anatomy. A. A viral vector expressing channelrhodopsin (ChR2) was injected into the medial septum (MS) at postnatal (P) day 7. Following a series of 25 flurothyl-induced seizures between P9-P13, pups were then subjected to intermittent MS-induced theta activity of 7 Hz during the critical period (P21-P25). Half the pups with early-life seizures (ELS) and controls (CTL) received either active blue light (BL) or inert yellow light (YL) during the critical period of spatial cognition. The rats were then studied as adults in the active avoidance task and had EEGs done. B. MS (pink) and ventral hippocampus (light green) in three-dimensional view. Red arrows represent horizontal plane and green and blue arrows represent vertical plane. Circled A and P denote anterior and posterior directions. Bottom: Two-dimension coronal view of interconnections of MS and hippocampus. In the MS, cholinergic (yellow circle), glutamatergic (pink circle) and GABAergic neurons (blue circle) form a highly interconnected local network. Neurons of these three classes project to glutamatergic pyramidal neurons and different subsets of GABAergic neurons in CA1. GABAergic neurons project back to the MS and form a feedback loop between the two anatomical distant brain regions.

The study design and timeline are provided in Fig. 1A. ELS were induced using a flurothyl solution dispersed by evaporation within a closed container. To artificially regulate hippocampal oscillations, we used pan-neuronal channelrhodopsin-2 (Chr2) medial septum (MS) stimulation. The MS, a midline structure that projects bilaterally to the hippocampus, was chosen based on our previous experiments using MS stimulation to precisely regulate hippocampal oscillations (Kloc et al., 2020). The septo–hippocampal pathway calibrates CA1 network excitability to different behavioral states and is crucially involved in theta rhythmogenesis (Müller and Remy, 2018). Furthermore, optogenetic stimulation of medial septal GABAergic neurons consistently modulates oscillations across multiple hippocampal locations in control and epileptic conditions (Hristova et al., 2021). In the MS, cholinergic, glutamatergic and GABAergic neurons form a highly interconnected local network (Fig. 1B). Neurons of these three classes project to glutamatergic pyramidal neurons and different subsets of GABAergic neurons in CA1. GABAergic neurons project back to the MS and form a feedback loop between the two anatomical distant brain regions. The major projection target of the MS is the hippocampal complex through the fimbra-fornix pathway, mainly the hippocampus proper and the entorhinal cortex, with fewer efferents in perirhinal and postrhinal cortices (Solari and Hangya, 2018; Gulyás et al., 1999; Kondo and Zaborszky, 2016). The MS also sends a limited number of axons to the medial habenula (Qin and Luo, 2009) and to retrosplenial, infra-limbic and prelimbic cortices (Gaykema et al., 1990; Unal et al., 2015).

After showing that MS optogenetic stimulation regulates hippocampus oscillations in both ELS and controls (CTL), pups received either 3 h of optogenetically-induced blue light (BL) intermittent stimulation or inert yellow light (YL) intermittent stimulation for 5 days during the critical period for memory development (P21–25). To determine whether stimulation resulted in long-term effects, rats were tested at P50–60 for spatial memory in the active avoidance task. EEG recordings were obtained within five days (P61-P65) of completion of the active avoidance testing. Following completion of the EEG recordings, animals were sacrificed for detailed histological examination of the MS and hippocampus.

### Viral Injection:

At P7, rats were injected with a viral vector expressing ChR2 into the MS as previously described (Kloc et al., 2020). Under isoflurane anesthesia, the skull was exposed, and a burr hole was placed in the skull (AP = 0.7 mm; ML = 0.0 mm) that allowed access to the MS with a Hamilton injection syringe. The needle was lowered 6.0 mm into the brain. A total of 0.8 μl of an adeno-associated virus expressing humanized ChR2 fused to EYFP driven by human Synapsin I promoter for optogenetic activation (AAV2-hSyn-ChR2(E123A)-EYFP; 5.7 × 10^12^ virus molecules/ml) (UNC Vector Core, Chapel Hill, NC) was injected slowly (0.1 μl/min) into 5 sites with the syringe raised by 0.2 mm before each injection (0.1 μl, 0.2 μl, 0.2 μl, 0.2 μl and 0.1 μl along the dorsal/ventral axis). The scalp was closed with sutures and the pups returned to their dam once they were ambulatory.

### ELS induction:

ELS animals received 25 flurothyl-induced seizures over 5 days, from P9–13. The method used was described in other papers from our laboratory (Karnam et al., 2009a; Holmes et al., 2015; Niedecker et al., 2021; Karnam et al., 2009b; Zhao and Holmes, 2006). All pups were placed in an octagon-shaped plastic container set in an airflow hood. Each pup was positioned in their own wedge of the octagon, facing the open central portal of the container. Flurothyl solution (Bis[2,2,2-trifluoroethyl] ether, 98% pure; Sigma-Aldrich) was loaded into a 1.0 ml syringe, and doses of approximately 0.02 ml were injected by hand onto filter paper in the central portal of the container at 2 min intervals, with each dose separated by 2 min. The flurothyl evaporated, was inhaled by the pups, and caused seizures. Behavioral features of the seizures were stereotyped and consisted of myoclonic jerks, followed by forelimb clonus, wild running, loss of posture and severe tonic posturing. Pups were removed from the flurothyl chamber upon tonic extension of both forelimbs and hindlimbs and placed in a holding container. The seizure typically ended within 30 s of removal from the flurothyl chamber. Within 5–10 min of the end of the seizure, the rats were upright and walking in the cage and within 30 min they were behaving normally. The pups reunited with the dam once they were upright and walking. Seizure-induction was spaced every hour, starting with the initial flurothyl exposure. CTL animals experienced no seizures but separated from dams for a similar amount of time (~ 30 min) as ELS animals, to control for maternal deprivation stress.

### Hippocampal and MS Implants:

At P18, 10 days following injection of the virus and two days after the last seizure, custom-made electrode arrays were implanted in both the MS and the CA1 region of the dorsal hippocampus. The MS implant included an optic fiber with two 50 μm diameter stainless steel EEG electrode recording electrodes glued to the surface that extended 0.25–0.5 mm from the end of the optic fiber. A 230 mm multimode optic fiber (Thorlabs, CFLC230–10; Montreal, Canada) channeled light stimulation to the MS septum. The percent light transmittance through the fiber was tested using light generated from Spectralynx LED source (Neuralynx, Montana) and measured by a light meter with a photodiode sensor (Thorlabs; Model PM100D). Using a 50 mm patch cable for testing, only fibers that allowed >70% light transmittance at approximately 0.5 mm from the tip of the optical fiber were used in the implants. Two CREE (CREE; Durham, NC) LEDs were used to transmit BL (BL = 465 nm; Flux = 35.2 lm) or YL (YL = 606 nm; Flux = 80.6 lm). The optical fiber was then glued to a 230 μm ferrule (Thorlabs, CFLC230–10; TP01235931). The optical/recording ensemble was lowered into the MS along the same path previously taken by the Hamilton injection syringe to a final depth of 5.8 mm below the brain surface. Using a custom connecter, LEDs were fastened to the ferrules set into the MS implant for stimulation via the Pulse program (Neuralynx, Montana).

The dorsal hippocampal implant consisted of three tetrodes of stainless-steel wires (50 μm) cut at an angle producing a range of electrode contacts spanning approximately 0.25 mm (California Fine Wire, CA, USA) were stereotaxically placed in CA1 in the left hippocampus (AP = −3.0 mm; ML = −2.5 mm; DV = 2.2 mm; bregma reference). Each wire was connected to custom gold Mill-Max pins (Neuralynx, Montana) that were set into the cap of the custom implant. Two skull screws (FHC Inc) were inserted, one screw was anterior to bregma with the other screw placed on the right side, mirrored to the hippocampal implant burr hole. Grounding was achieved via connection to the right cerebellar screw and a signal reference wire (50 μm diameter) placed at the brain surface over the cerebellum. Both implants were fixed to the skull via the skull screws and Palacos bone cement (Heraeus Medical, USA). Following suturing, a topical antibiotic was applied. The interval between surgery and the beginning of electrophysiological recordings and stimulation was four days.

### Optogenetic Stimulation:

At P21, rats were tested for MS optogenetic regulation of hippocampal oscillations. Rats were placed in a 41 cm high ceramic flowerpot that was 18 cm wide at the base and lined with home cage bedding and connected to a custom-made adapter for the Neuralynx head stage with preamplifiers and a fiber optic optogenetic light-emitting diode (LED) driver (Neuralynx, Montana, USA). The purpose of the flowerpot was to limit animal movement without restraint to measure hippocampal oscillations while minimizing potential sources of stress.

The pulse program (Neuralynx, Montana) was used to stimulate YL or BL LEDs. Maximum light intensity was set to 100%. Sinusoidal wave stimulatory patterns were generated using the Pulse2 program which generated current for the LEDs via the FLED driver (Neuralynx, Montana). The stimulation frequencies of artificial sinusoidal waves at frequency during the 5–12 Hz ramp (periodicity = 1/Frequency) were generated by creating an ascending and descending light intensity gradient with a mean light intensity of 52%. The light stimulation intensity was divided into 23 epochs corresponding to 0–255 bits where each increment was a percentage of peak amplitude at 255 bits (i.e., ranging from 1.6 to 100%).

To assess the response of the hippocampus to MS stimulation, a “ramp” protocol was used with optogenetic stimulations administered for 60s, sequentially from 5 to 12 Hz, with 15 s of rest between frequencies. The EEGs were visually inspected to determine if there was optogenetic regulation of the EEG. The rats were then stimulated with either BL or YL at 7 Hz in a 2 min stimulating, 2 min off loop for 3 h a day for five days (P21-P25). All records were subsequently analyzed using density spectral analysis to confirm presence or absence of theta regulation. Because we could only stimulate one rat at a time, the stimulation occurred between 08:00 to 20:00 with the lights on. In each group of stimulated rats, the rats were stimulated in the same order over the five days. During daily experimental stimulations, rats were predominately awake, although sleep was occasionally noted.

### EEG Recordings:

Rats were tethered to an electrophysiology cable during recording sessions. Signals were pre-amplified X1 at the head-stage interface with the custom implant mill-max pins. Signals were channeled through the tether cable to the signal amplifiers and computer interface and amplified to ±1 mV. Sampling frequency of EEG was at 30.3KHz and filtered at 1–9000 Hz (Neuralynx) and subsampled offline at 1000 Hz. Both MS and intra-hippocampal EEGs were referenced against the cerebellar electrode.

All EEG analyses were performed offline using BESA^®^ software (Gräfelfing, Germany). Spectral power, coherences and phase locking were obtained for each rat at each optogenetic stimulation frequency using methods previously described in our laboratory (Kloc et al., 2020; Mouchati et al., 2020).

### Spectral power:

After a single taper with the Hamming windowing function, the fast Fourier transform (FFT) of the EEG was calculated from 0 to 100 Hz using a sliding window (window = 1 s, overlap = 0.5 s). Waveform frequencies were classified as follows: delta (0–4 Hz), theta (5–12 Hz), slow gamma (30–50 Hz) and medium gamma (70–90 Hz).

### Coherence:

Coherence is a measure of the linear relationship between two signals at a specific frequency. Coherence is defined as the normalized cross-spectrum: C(*f*) = 〈*Sxy*(*f*)〉√〈*Sxx*(*f*)〉〈*Syy*(*f*)〉, where *Sxy* is the cross-spectral density between two signals, and *Sxx* and *Syy* are the autospectral densities for signals *x* and *y*, respectively. Coherence is defined as the magnitude of coherence: Coh(*f*) = |C*xy*(*f*)| = |〈*Sxy* (*f*)〉√〈*Sxx*(f)〉〈*Syy*(*f*)〉|.

The estimated coherence for a given frequency ranges between 0 and 1. A value of 0 indicating that the two signals are totally uncorrelated, and a value of 1 indicates perfect correlation. Coherence is a bivariate measure, which means it considers only two signals simultaneously. It is also a nondirectional connectivity measure, that is, Coh(*f*) = Coh*yx*(*f*). Both intra-hippocampal and MS-hippocampal coherences were calculated.

### Phase locking value (PLV):

PLV is a measure for phase synchronization. The PLV is defined based on the average of phase angle difference between two signals over time at a specific frequency (Lachaux et al., 1999): PLV*xy*(*f*) = |1*n*Σ*ei*•(Φ*x,t* − Φ*y,t*)*nt* = 1|, where *n* is the number of time points, *i* is the imaginary number that results from taking the square root of −1, and Φ*x* and Φ*y* are phase angles from signals *x* and *y* at frequency *f*.

The PLV takes values between 0 and 1. A value close to 1 means perfect phase locking, while a value close to 0 results from a random phase distribution over time. Like coherence, PLV is a nondirectional connectivity measure; that is, PLV(*f*) = PLV*yx*(*f*). Intra-hippocampal and MS-hippocampal coherences were calculated.

The coherence and PLV provide similar results in many cases. However, coherence is influenced by strong increases or decreases in power as the analysis includes power information of the signals. For instance, if connectivity increases, but the amplitudes of signals simultaneously decrease, estimated coherence values may be biased. PLV is insensitive to the amplitudes of signals and only depends on the phase relationship between two signals.

### Active avoidance:

Animals underwent testing in the active place avoidance task (Biosignal; Brooklyn, New York) at P50-P60. In this task, animals must attend to their ever-changing position in the room frame lest they be rotated into a pre-determined non-marked zone where they receive a mild electrical shock (Barry et al., 2015; Kloc et al., 2020; Niedecker et al., 2021; Barry et al., 2020; Barry et al., 2016a; Barry et al., 2016b).

One day prior to the active avoidance task, rats were anesthetized and implanted with a stainless-steel swivel in the skin between the shoulders. The swivel was attached to a cable with an LED at the end allowing for automated tracking and the delivery of shocks.

The arena consists of a steel disc 82 cm in diameter lighted from both above and below. The arena is centered in a room where it is approximately 50 cm from black curtains on the S and E sides and 50 cm from white walls on the N and W sides. The N and W walls have an 11 cm gray power-strip that forms a continuous line 50 cm above the floor of the arena. Two rectangular spatial cues (30cmX43cm) depicting a red star (centered at W position) and a black circle (centered at N position), both on a white background, were placed in the arena 18 cm above the floor. An additional rectangular polarizing cue (53cmX84cm) made of white paper with five 2.5 cm wide diagonal black stripes was centered at the N position, 5 cm above the gray power strip. The cues remained constant throughout the testing period.

On the first day of training, the animal was connected to the shock cable and placed in the rotating arena for a 10 min habituation period without shock. On all subsequent sessions, rats received a 0.4 mA shock in an unmarked 876cm^2^ wedge-shaped sector covering a 60° arc in the NE sector of the arena. The shock zone was stable in the room frame while the arena rotated 360° per minute. The entrance latency of the shock was 1 ms, the shock duration was 0.5 s and the inter-shock latency was 2 s. Rats were trained in eight 10 min sessions per day for 2 days (16 sessions). Outcome variables for the test include number of entrances into the shock zone per trial and number of shocks per trial. Rats were arbitrarily designated as learners (a mean of 10 shocks or less over the last four trials) and non-learners.

Fig. 6 provides examples of activity maps demonstrating position of the rat during the 12th active avoidance trial. Fig. 6 Ci and 6Ciii show examples of maps from learners while Figure 8Cii and 8Civ show maps from non-learners.

### Post Active Avoidance EEG:

Following active avoidance, rats had an awake EEG recording while in the ceramic flowerpot (P61–65). The EEGs were analyzed for hippocampus theta frequency, relative and absolute spectral power, coherence and PLV.

### Tissue Processing and Imaging:

Following all experiments, rats were deeply anesthetized and perfused with ice-cold PBS followed by 4% paraformaldehyde. Brains were removed and postfixed for 24–48 h, then incubated in 30% sucrose until fully impregnated. Slide-mounted sections (40 μm thick) were taken from the MS and hippocampus on a cryostat. Slides were permeabilized with 0.5% Triton X-100 in PBS and blocked with 10% fetal goat serum prior to overnight incubation with rabbit anti-NeuN primary antibody (1:500; ThermoFisher Scientific, Massachusetts, USA). Following primary antibody incubation, slides were incubated in a FITC anti-rabbit secondary antibody (Jackson ImmunoResearch, Pennsylvania, USA) and cover-slipped with DAPI-containing mounting medium (Invitrogen, California, USA). All images were obtained with a Nikon C2 laser scanning confocal microscope (Nikon, Tokyo, Japan). Images were processed using FIJI/ImageJ (NIH).

### Image Analysis:

Image processing was performed blinded to the stimulus condition. In tissue sections containing the hippocampus, cell counts were obtained manually in FIJI/ImageJ – the density of DAPI and NeuN-labeled neurons led automated counting tools to be inaccurate. Individual NeuN- and DAPI-labeled structures were visually identified by the experimenter based on shape, size and presence of defining dark pixels denoting unlabeled extracellular space. The CA1 pyramidal cell layer was outlined by a region of interest (ROI), which was consistent across all tissue sectioned (0.23 ± 0.002 mm^2^). For tissue sections containing the MS, a separate group of consistently sized ROIs were used to normalize neurons and total cells counted from the MS (2.14 ± 0.23 mm^2^). In the MS ROI, NeuN and DAPI-labeled structures were clearly defined and therefore the plugin “Analyze Particles” was used to count both neurons and total cell nuclei (minimum shape detection size 20μm^2^). All automated counts were visually verified for accuracy of the analysis by the experimenter. One slice of high-quality staining without electrode damage was used from the dorsal hippocampus (CA1) and MS for each rat. Cell counts were obtained manually in the hippocampus and using the “Analyze Particles” plugin in the MS (all automatic counts were verified by the experimenter for accuracy). Total cell counts in each CA1 and MS ROI included in the analysis were normalized by measurement area to account for minor variations in ROI shape/size. Cell counting was done by investigators blinded to the experimental group. Three investigators (ML, CD, CAS) were involved with the cell counting and at least 10 specimens were counted by the same three people to ensure consistency and reliability of the counting techniques.

### Sample size/power calculations and statistics:

The primary outcome goal was to address the hypothesis that 7 Hz optogenetic stimulation following ELS during the critical period (P21–P25) would result in long-standing improvement, whereas 7 Hz optogenetic stimulation in CTL rats without ELS would have no discernible effects on spatial cognition. For sample size calculations, number of entrances into the shock zone was used as the primary outcome measure. Based on results from a recent study with a similar study design where the standard error for mean entrances into the shock zone per trial was approximately 2.5 (Kloc et al., 2020), a sample size of 7 rats per experimental condition (ELS-BL, ELS-YL, CTL-BL, CTL-YL) was predicted to detect a 20% (90% power, 5% α) difference in number of entrances into the shock zone over the 16 trials.

For all EEG measures between 5 and 10 channels of hippocampal EEG and 2 channels of MS EEG devoid of artifact were averaged for each rat. As expected, the coherence and PLV measures were high in the intra-hippocampal CA1 electrodes. EEGs obtained during the ramp stimulations (light off) were evaluated for coherence. The mean ± standard deviation (SD) from 768 coherence measurements from CTL (*n* = 20) and ELS (*n* = 22) rats were calculated for total (0.903 ± 0.113), delta (0.801 ± 0.173), theta (0.868 ± 0.137), slow gamma (0.929 ± 0.109) and med. Gamma (0.906 ± 0.124). The value of 0.6 for coherences and PVL was 2 SD below the mean for all frequencies except delta where 0.6 was greater than 1 SD below the mean. Because of the high coherences in the intra-hippocampal electrodes, coherences <0.6 were presumed to be coming from electrodes not in the cell layer. Channels with a coherence value <0.6 in any of the frequencies were not used for any additional analyses. Supplementary Figs. 1A, 1B provided examples on how electrode channels were discarded. Most channels were eliminated because of electrode artifact. As shown in Supplementary Figs. 1A, 1B, for the most part electrode pairs with low coherences were also low in voltage. Supplementary Tables 1 and 2 list electrodes used and discarded in the ramp (Supplementary Table 1) and post-active avoidance EEGs (Supplementary Table 2). Because of electrode degradation during the period between the ramp studies and studies done after active avoidance, fewer electrodes were available for analysis in the post-active avoidance testing. There were no statistical differences between mean electrodes used in each group.

For the ramp study, epochs of EEG before and during MS optogenetic stimulation were assessed for relative power, absolute power, coherence and PLV at frequencies of 5–12 Hz using the paired *t*-test. Post active avoidance EEG parameters and cell counts from the histological specimens were compared using ANOVA with Tukey’s multiple comparison test to assess differences among the four groups.

A 2-way ANOVA for two independent variables (shocks or entrances vs trials) in the active avoidance test was used to determine differences between the ELS and CTL groups receiving BL or YL when group size was equal. When group size was not equal, a mixed-effects analysis was performed. Data is presented as means±standard error of the mean (SEM). Depending on the presentation of the data, error bars were either placed above, below or in both directions at the mean. Significance was set a *p* < 0.05 and a false detection rate was assessed using the Holm-Sidak statistic and only corrected *p* values are presented. All statistical analyses were done using GraphPad Prism (version 9.4.1, GraphPad Software, San Diego, California USA),

## 3. Results

The total number of rats initially started in the study was 70. Sixteen pups died during injection of the viral vector or hippocampal electrodes; three pups during flurothyl-induced seizures induction and nine pups were not used due to technical issues with the recordings. The final group size (*n* = 42) with evaluable data was ELS-BL (*n* = 11), ELS-YL (n = 11), CTL-BL (*n* = 10) and CTL-YL (n = 10), which exceeded the minimum number of rats indicated by sample size calculations.

### 3.1. Optogenetic BL MS stimulation results in regulation of hippocampal oscillation frequency in both ELS and CTL rats

**Fig. 2.**
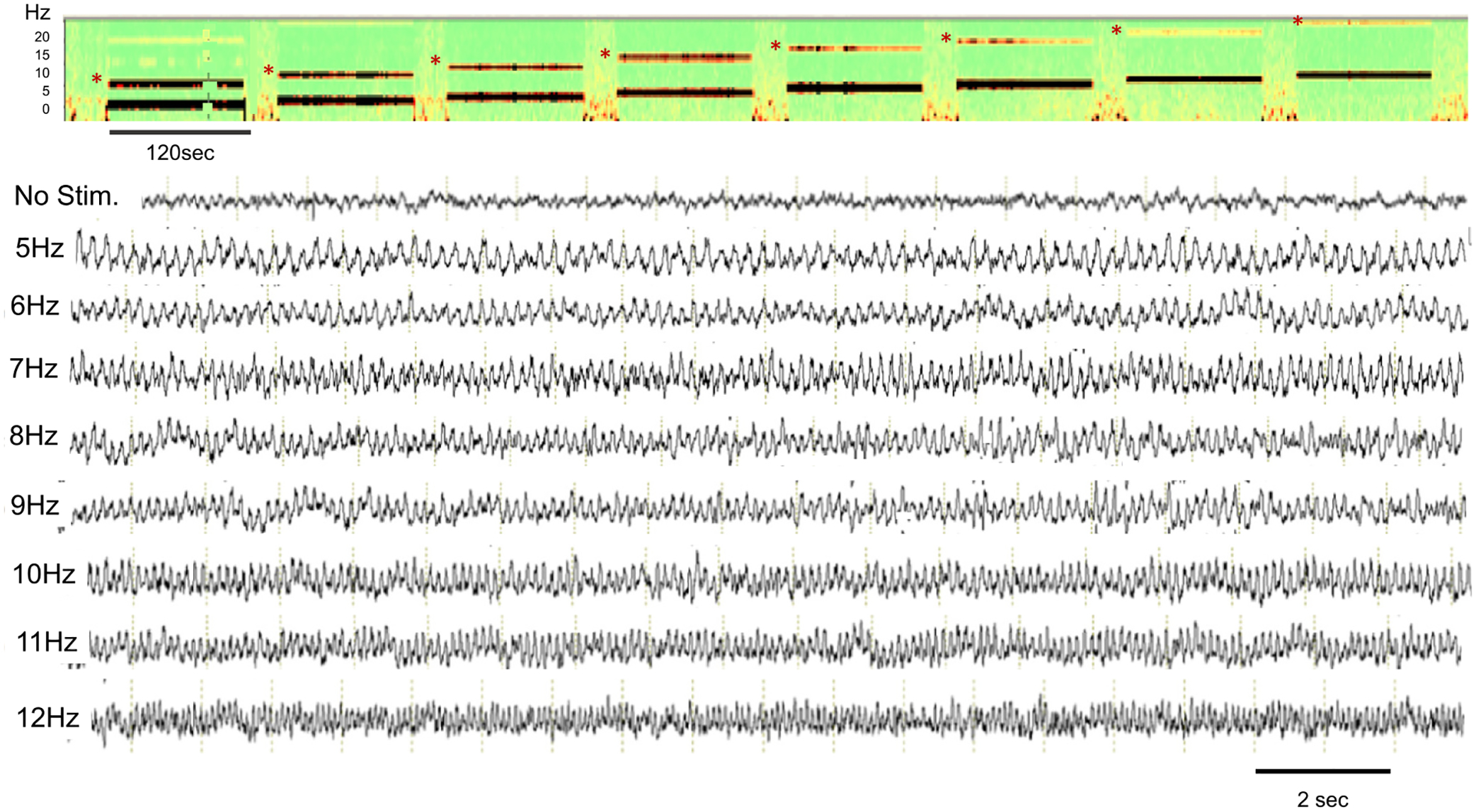
Density spectral array and examples of EEGs from CA1 of the hippocampus. The one-to-one relationship between MS stimulation and hippocampal theta frequency is apparent. The density spectral array (top) is from rat pup undergoing MS stimulation from 5 to 12 Hz delivered sequentially (“ramp” stimulation). Note the harmonics (*) at all stimulation frequencies. The bottom unfiltered EEG traces at baseline and during the ramp stimulations from 5 to 12 Hz. Note the timeline differs in the density array and the EEG tracings.

Optogenetic stimulation of the MS resulted in clear frequency-matched EEGs in CA1 of the hippocampus. As shown in Fig. 2, stimulation at 5–12 Hz resulted in regulation of on-going hippocampal oscillations by the optogenetic stimulation, i.e. MS stimulation at 8 Hz resulted in 8 Hz theta oscillations. While the MS stimulation frequency dominated the EEG pattern, intermixed EEG at different frequencies were found. Harmonics were noted with each of the stimulation frequencies. Harmonics have been described previously with this experimental design (Kloc et al., 2020; Mouchati et al., 2020) and are a Fourier decomposition of the waveform, rather than originating in a separate neuronal process (Laxpati et al., 2014) and will therefore not be discussed.

To assess frequency-matched CA1 EEG by MS stimulation coherences and PLV between MS electrodes and CA1 electrodes were compared in the CTL and ELS BL groups at each stimulation frequency. As expected, coherences and PLV were significantly higher during epochs of BL stimulation compared to non-stimulation epochs in both the ELS and CTL rats (Supplementary Fig. 3). This finding supports previous work from our laboratory showing that optogenetic stimulation of the MS resulted in high coherences between the MS and hippocampus at each stimulation frequency (Kloc et al., 2020).

**Fig. 3.**
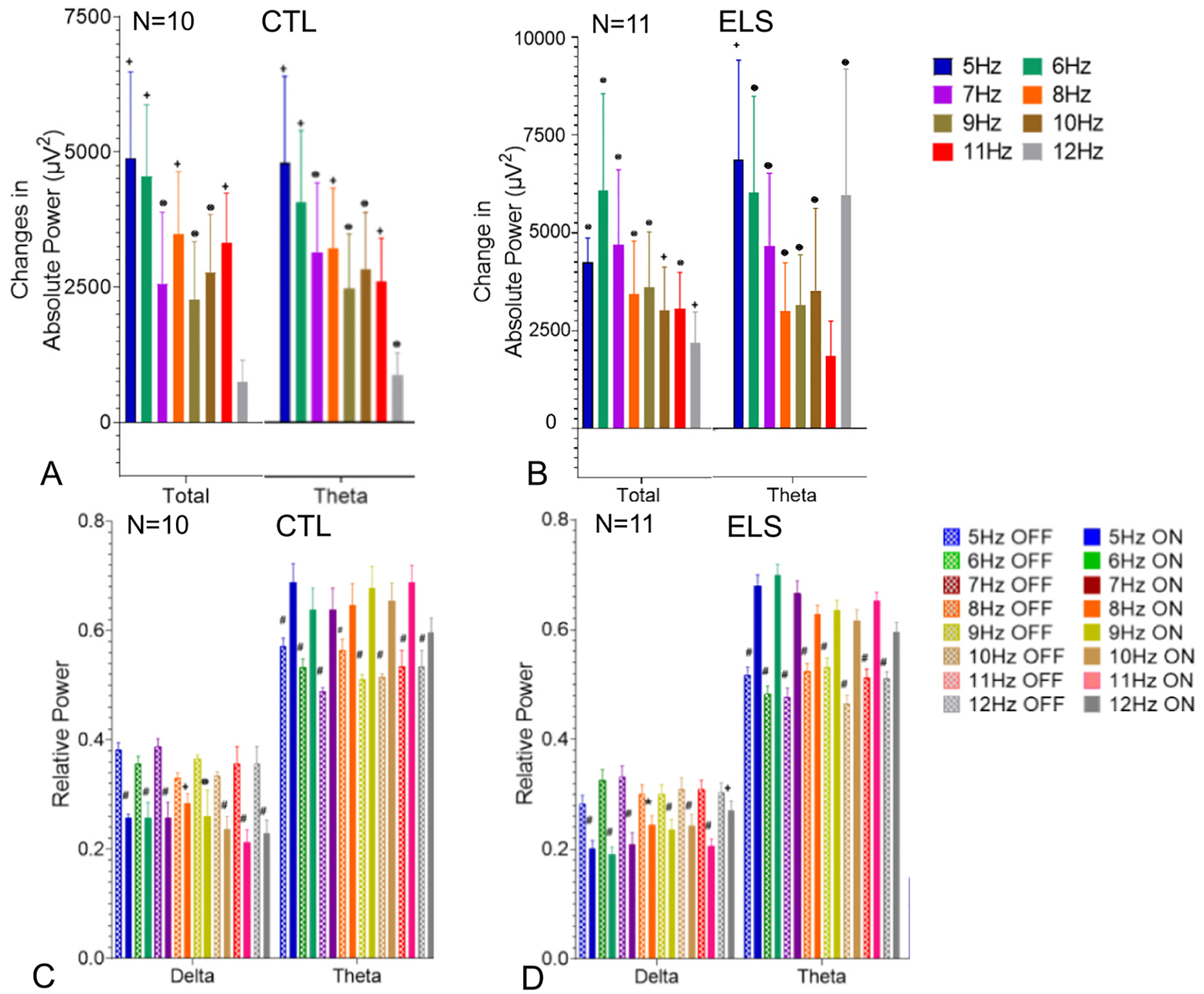
Effects of MS 5–12 Hz optogenetic stimulation on CA1 EEG absolute power in CTL-BL (A) and ELS-BL (B) and relative power in CTL-BL (C) and ELS-BL (D). The graphs show the change in absolute power and relative power from pre-stimulation EEG to MS optogenetic stimulation from 5 to 12 Hz in total (delta + theta + slow gamma and med. Gamma frequencies) and theta. and changes in In both the CTL (A) and ELS (B) groups there was an increase to total and theta absolute power with stimulation from 5 to 12 Hz. In both the CTL (C) and ELS (D) groups there was a decrease in relative delta and increase in relative theta. Data from all bands are shown in Supplementary Figs. 4, 5. (*p* values: ⊗ ≤0.05; + ≤0.01; * ≤0.001; # ≤0.0001).

**Fig. 4.**
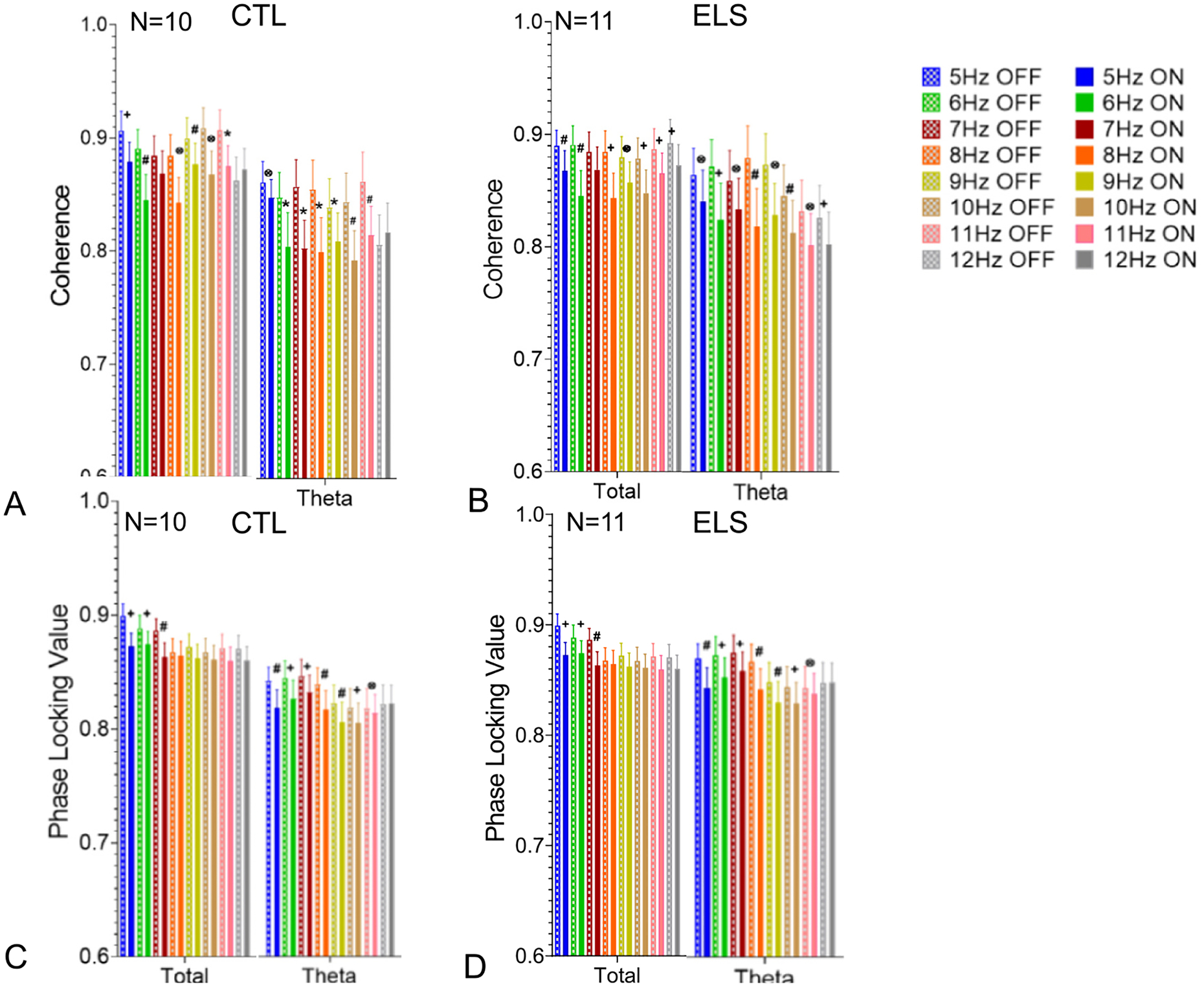
Effects of MS 5–12 Hz optogenetic stimulation on CA1 EEG coherence in CTL-BL (A) and ELS-BL (B) and PLV in CTL-BL (C) and ELS-BL (D). Pre-stimulation (“OFF”) followed by MS optogenetic stimulation (“ON”) for each frequency from 5 to 12 Hz. In both the CTL (A,C) and ELS (B,D) groups decreases in coherence and PLV were seen in total and theta bands. Data from all bands are shown in Supplementary Figs. 6, 7. (p values: ⊗ ≤0.05; + ≤0.01; * ≤0.001; # ≤0.0001).

To determine if there were any differences in the effect of MS stimulation on hippocampal EEG, 15 s epochs during each stimulation frequency in the theta band (5–12 Hz) were analyzed. Dorsal hippocampal absolute and relative power, coherence and PLV values in total (delta, theta, slow gamma, med. gamma) and delta, theta, slow gamma and medium gamma were compared (Figs. 3–4). For visualization purposes, Figs. 3–4 include only the total and theta band values. All bands studied are available in Supplementary Figs. 3–6. Absolute power showed marked increases in total and theta power in both the ELS-BL and CTL-BL MS stimulation groups (Fig. 3A,B/Supplementary Fig. 3). In both the CTL and ELS rats, BL MS stimulation resulted in decreased relative power of delta, medium gamma and slow gamma and increased relative theta power in CA1 (Fig. 3C,D/Supplementary Fig. 4).

As expected, coherences and PLV between the tetrodes in the hippocampus were quite high since all the electrodes were in the CA1 region, albeit at different depths. Both intra-hippocampal coherence and PLV were decreased at multiple bands with BL MS stimulation in both the CTL and ELS groups (Fig. 4/Supplementary Figs. 5, 6). There was a strong relationship between stimulation frequencies that reduced coherence with those that reduced PLV. It is known that intra-hippocampal coherences in theta and gamma vary across the CA1 (Caixeta et al., 2013; Buzsáki et al., 1986). In a laminar analysis of coherence during slow wave activity in behaving rats, a gradual shift in phase occurred with increased depth of the electrode in CA1 (Buzsáki et al., 1986). Since all electrodes were in CA1 at various depth, the finding supports existing evidence that theta is produced by several rhythmical dipoles along the somatodendritic surface of CA1. The findings suggest that MS generated theta intrudes upon endogenous theta resulting in a decrease of coherence and PLV within the theta band.

These results show that optogenetic MS stimulation results in robust changes in power, coherence and PLV in the EEG of the CA1 region of the hippocampus during the MS stimulation. Optogenetic stimulation control of the CA1 EEG appears similar in ELS and CTL rats. Optogenetic stimulation with YL had no effect on any EEG features.

**Fig. 5.**
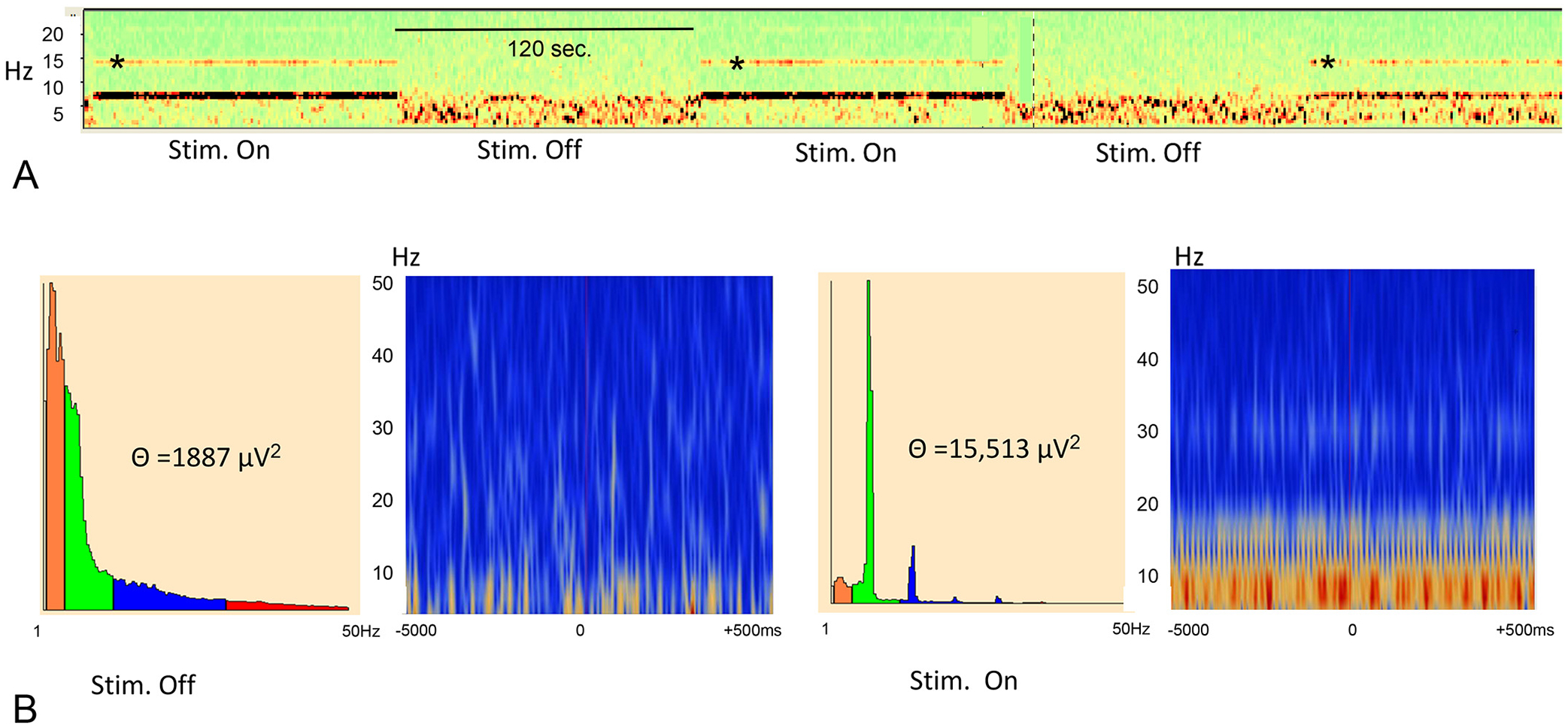
Effects of 7 Hz MS stimulation on CA1 EEG. A. Density spectral array showing 7 Hz band during stimulation on (120 s) and during stimulation off (120 s) from a single EEG channel. The thick band is at 7 Hz with harmonics demonstrated. B. Power spectrum (left panel) and time-frequency display for 7 Hz stimulation off (left) and stimulation on (right) from a single channel. Time frequency displays were generated by averaging power over trials. With 7 Hz MS optogenetic stimulation there was a marked increase in theta power with time-frequency domination by 7 Hz activity. The panel is colour coded with the brighter colors demonstrating higher theta power.

As seen during the assessment of MS stimulation with escalating frequency, the intermittent 7 Hz stimulation resulted in clear regulation of the CA1 EEG with increases in relative theta power with a well-defined time-frequency modulation of theta EEG during the 2 min stimulation periods (Fig. 5). No behavioral changes were seen during either the BL or YL daily stimulations.

### 3.2. Active avoidance

**Fig. 6.**
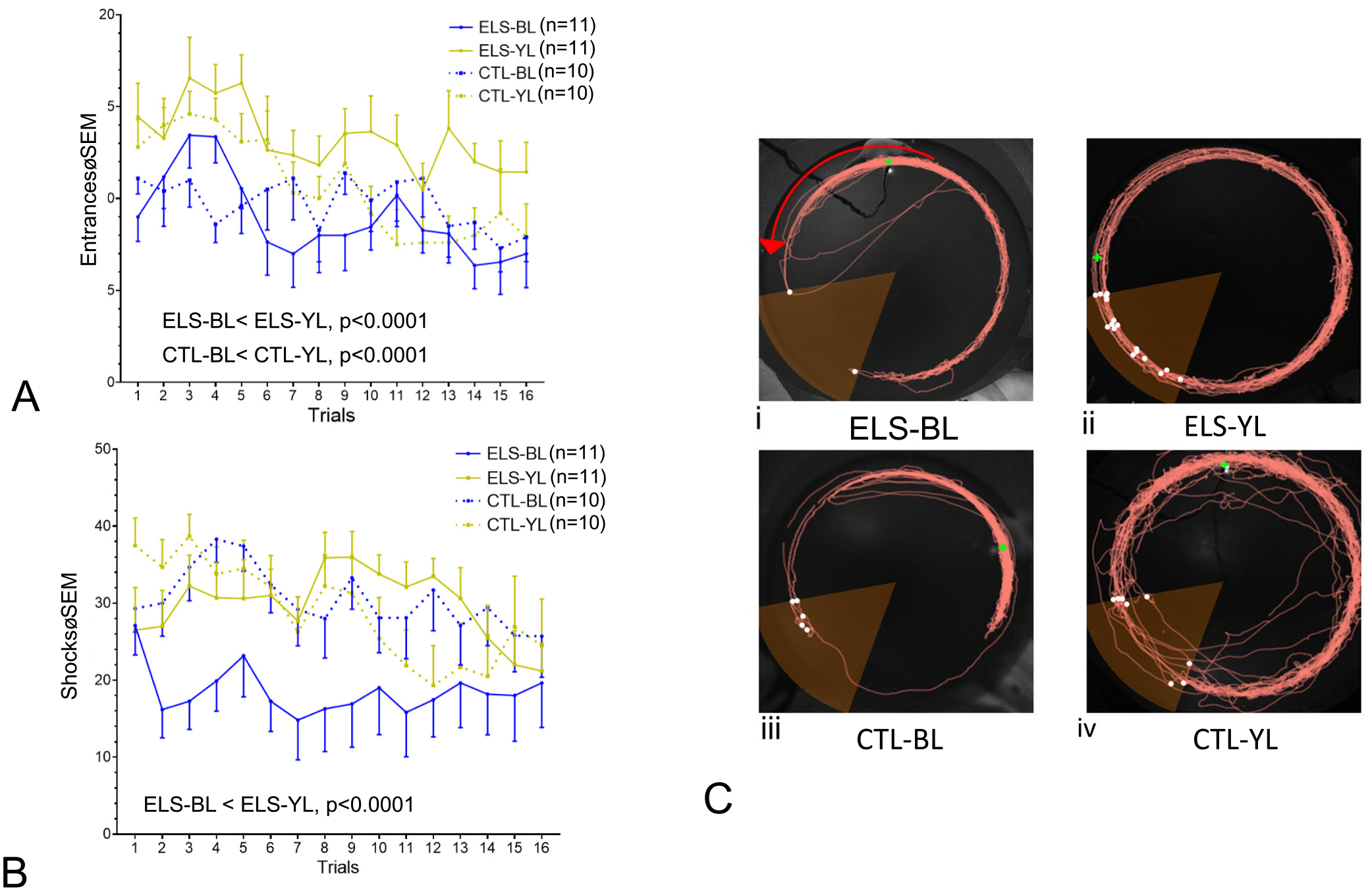
Comparison of mean entrances (A) and shocks (B) per trial in the four groups of rats. A. Group differences were noted in entrances per trial with both the ELS and CTL groups having fewer entrances into the shock zone than the YL treated groups. B. Group differences were noted in the number of shocks with the ELS-BL group receiving fewer shocks than the ELS-YL group. The primary statistical findings are noted in both graphs. C. Examples of activity maps demonstrating position of the rat during the 12th trial. ELS-BL (i), ELS-YL (ii), CTL-BL (iii), CTL-YL (iv). White dots represent shocks; the green dot shows position of the rat at the end of the trial. Red arrow shows direction of the rotating arena. Note reduced time in the shock zone in ELS-BL (i) and CTL-BL (iii) compared to the other groups.

In the mixed-effects model, simple main effects analysis showed significant decreases in entrances across trials in all groups (F = 21.18, *p* < 0.0001) and significant differences between groups in entrances per trial (F = 7.828, *p* = 0.0002) (Fig. 6A). In the ELS group, the BL group had significantly fewer entrances than the YL group (F = 20.39, p < 0.0001). Likewise, in the CTL groups, the BL had significantly fewer entrances than the YL group (F = 23.74, p < 0.0001). There were no differences in the entrances between the ELS-BL and CTL-BL and CTL-YL or differences between the CTL-BL and CTL-YL.

In the mixed-effects model, simple main effects analysis also showed significant decreases in shocks across trials in all groups (F = 11.31, p < 0.0001) with significant differences between groups in shocks per trial (F = 12.61, p < 0.0001) (Fig. 6B). The ELS-BL group had significantly fewer shocks per trial than the ELS-YL (F = 21.19, p < 0.0001), CTL-BL (F = 51.08, p < 0.0001) and the CTL-YL (F = 22.88, p < 0.0001) groups.

Although there is a correlation between number of entrances and number of shocks (Pearson *r* = 0.1811; *p* = 0.0006), i.e., the more entrances the greater the number of shocks, entrances appear to be a better measure of learning than shocks. Some animals made no attempts to move out of the shock zone and therefore received multiple shocks per entrance, whereas other animals escaped the shock zone immediately after getting their first shock. An example of this observation is in the tracing from the rat in Fig. 6Cii, where the rat entered the shock zone and did not attempt to escape.

In the ELS-BL group, there were 9 rats classified as learners (a mean number of 10 entrances or less over the last four trials) and 2 non-learners, whereas in the ELS-YL group, there were 5 learners and 6 non-learners, showing a non-significant difference in groups (Fisher’s exact test = 4.545, *p* = 0.089). In the CTL-BL group, there were 5 rats classified as learners and 5 as non-learners, while in the CTL-YL group there were 6 rats classified as learners and 4 as non-learners.

In the mixed-effects model significant differences were found in mean entrances (F = 3.742, *p* < 0.0001) across trials in the learners (*n* = 25) and non-learners (*n* = 17) (Supplementary Fig. 7). This shows that the criteria used to distinguish learners from non-learners was effective in distinguishing the groups.

### 3.3. Post active avoidance EEG

At P60, rats underwent EEG monitoring while awake in the flowerpot. Supplementary Table 2 lists number of electrodes used in each of the groups. The number of electrodes used was fewer than in the ramp studies due to electrode degradation. No differences were noted in mean number of electrodes used in each group. While rare interictal spikes were noted in the ELS animals, no electrographic or behavioral seizures were seen. There was a significant difference in theta frequency in the four groups (F = 3.623; *p* = 0.0212; ELS-BL: 7.632 ± 0.074; ELS-YL: 7.083 ± 0.172, CTL-BL 7.488 ± 0.135; CTL-YL: 7.292 ± 0.092). Tukey’s multiple comparisons test showed that the ELS-BL had a higher mean frequency than the ELS-YL group (adjusted *p* = 0.0321).

**Fig. 7.**
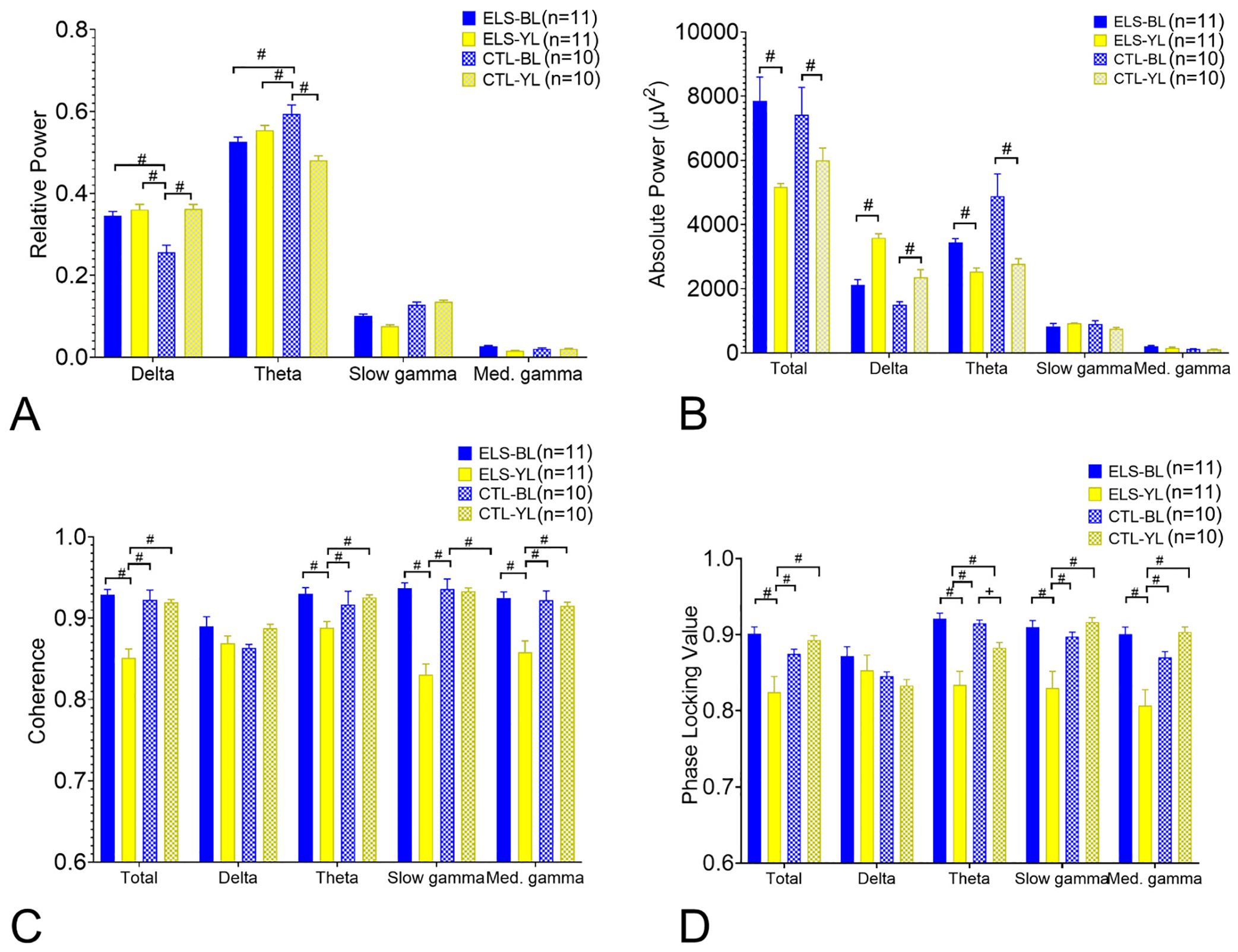
Comparison of EEG features following active avoidance in ELS and CTL rats. A. Relative power. The CTL-BL group had the lowest relative delta and highest relative theta compared to the other groups. B. Absolute power. In both the ELS and CTL groups, BL resulted in higher total power, lower delta power and higher theta power than the YL groups. C. Coherence. The ELS-BL had higher coherences in total, theta, slow gamma and med. Gamma coherences than the other groups. D. PLV. The ELS-BL had higher PLV in total, theta, slow gamma and med. Gamma than the ELS-YL groups. PLV was higher in the CTL-BL group than the CT-YL group. (p values: ⊗ ≤0.05; + ≤0.01; * ≤0.001; # ≤0.0001).

Significant differences were seen in relative power in the differences among the four groups in delta (F = 5.267; *p* < 0.0001) and theta (F = 8.483; p < 0.0001) (Fig. 7A). Post hoc testing with Tukey’s multiple comparison test showed the CTL-BL group with the lowest relative delta and highest relative theta compared to the other groups. In absolute power, significant differences were noted in total power across all bands (F = 23.34; *p* < 0.0001) and the delta (F = 27.77; p < 0.0001) and theta bands (F = 13.78; p < 0.0001) (Fig. 7B). Post hoc testing showed that with both the ELS and CTL groups, BL resulted in higher total power, lower delta power and higher theta power than the YL groups. These findings indicate that BL stimulation had little effect on relative power in rats with ELS but had significant effects on absolute power in both the ELS and CTL groups.

Significant differences were seen in total (F = 21.51; p < 0.0001), theta (F = 8.018; p < 0.0001), slow gamma (F = 34.82; p < 0.0001) and med. Gamma coherence (F = 10.84; p < 0.0001) (Fig. 7C). Post hoc testing showed that the ELS-BL had higher coherences in total, theta, slow gamma and med. Gamma coherences than the other groups. Likewise, with PLV, there were significant differences in total (F = 29.55; p < 0.0001), theta (F = 10.13; p < 0.0001), slow gamma (F = 34.53; p < 0.0001) and med. Gamma PLV (F = 37.12; p < 0.0001) (Fig. 7D). Post hoc testing showed that the ELS-BL had higher total, theta, slow gamma and med. Gamma PLV than the ELS-YL groups. PLV was higher in the CTL-BL group than the CT-YL group. As expected, the coherence and PLV measures paralleled each other and together demonstrate that rats with ELS receiving BL have higher functional connectivity than rats receiving YL. The findings provide evidence that in the ELS group, BL ameliorates the impact of seizures on coherence and PLV.

To assure that removing electrodes with coherences below 0.6 did not bias the results, we added these previously eliminated electrodes to the analysis. Significant differences in groups remained in total (F = 5.500; *p* = 0.0010), theta (F = 6.528; *p* = 0.0003), slow gamma (F = 6.966; *p* = 0.0001) and med. Gamma (F = 2.910; *p* = 0.0340) coherence. These results indicate that using a < 0.6 coherence threshold for electrode exclusion does not result in bias.

In previous work from our laboratory, we reported that coherence serves as a biomarker for spatial cognitive outcome post-ELS with higher theta coherences between the hippocampus and PFC associated with better performance in the active avoidance task (Niedecker et al., 2021). In an exploratory analysis we therefore compared coherence and PLV values in the intra-hippocampal electrodes in the learners and non-learners (Supplementary Fig. 8). Because of the small sample size, the data the data is presented in a box and whiskers plot. Differences were found between the learners and non-learners in both coherence and PLV. In the ELS group, differences in coherence were seen in total (F = 7.372; *p* < 0.0001), delta (F = 4.791, *p* = 0.0029), theta (F = 10.92, p < 0.0001), slow gamma (F = 12.34, p < 0.0001) and med. Gamma bands (F = 4.505, *p* = 0.0042) (Supplementary Fig. 8A). For the most part, Tukey’s multiple comparison test showed higher coherences in both the BL and YL group following ELS in the learners than the non-learners. Compared to the learners in the ELS-YL group, coherences were higher in all bands in the ELS-BL learners. Differences in PLV were seen in total (F = 4.042; *p* < 0.0082), delta (F = 3.276, *p* = 0.0223, theta (F = 14.93, p < 0.0001), slow gamma (F = 1 11.50, p < 0.0001) and med. Gamma (F = 6.555, *p* = 0.0018) (Supplementary Fig. 8B). PLV in both the BL and YL group following ELS were higher in the learners than the non-learners. As with coherence values, the PLV values in the ELS-BL learners were higher than the ELS-YL learners in the total, slow gamma and med. Gamma.

**Fig. 8.**
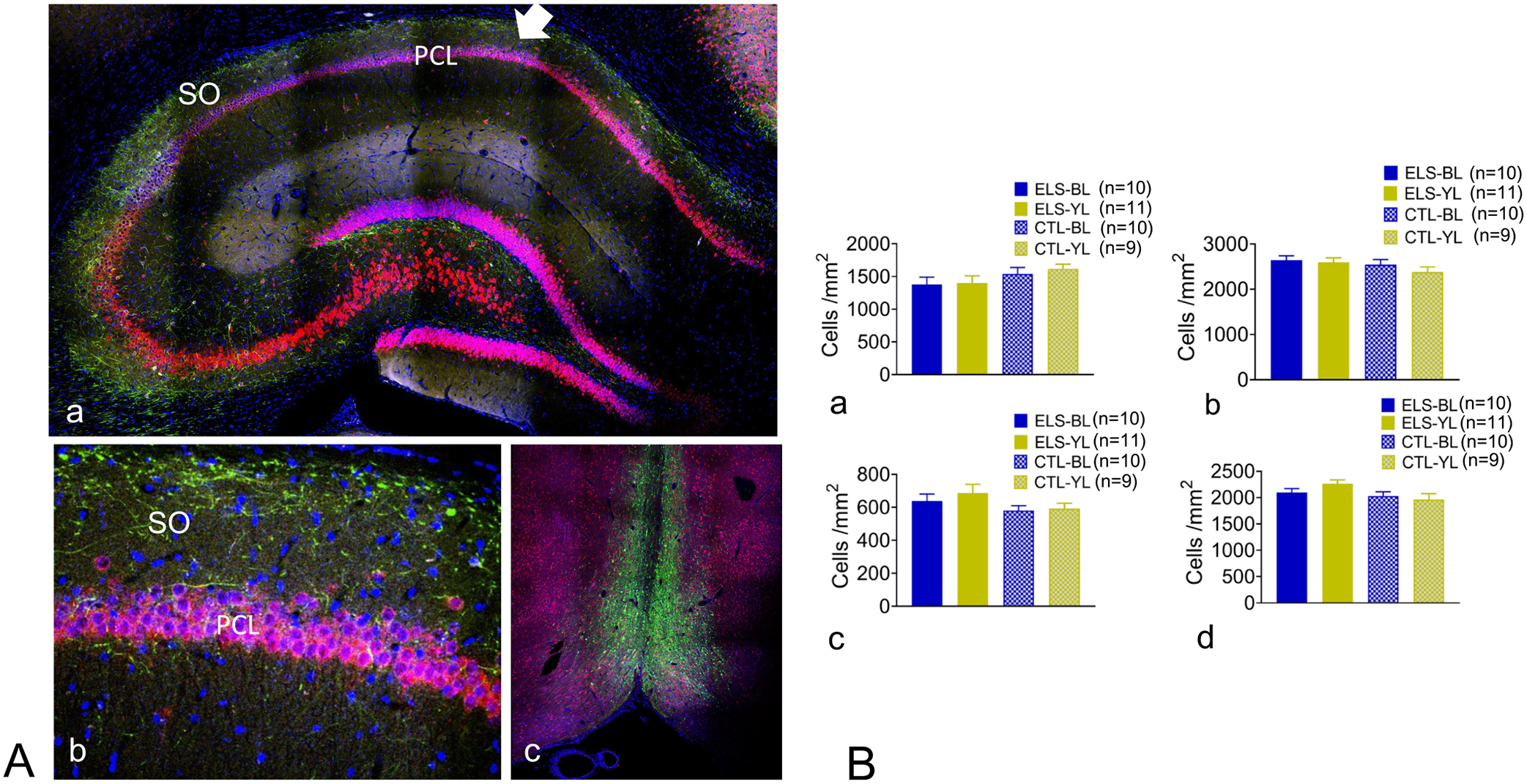
Histology and cell counts. A. Hippocampus contralateral to the implant showing Yellow Fluorescent Protein (YFP) (green) which is expressed by the viral vector and fused to the CHR2 protein, DAPI (blue), a non-cell specific stain of nuclei and NeuN (red) which stains mature neurons. Septal axons that are expressing ChR2-YFP (green) are seen throughout the projecting axons in the stratum oriens (SO, arrow) to the pyramidal cell layer (PCL). 20× magnification. B. Magnification of CA1 region showing axons in the stratum oriens innervating neurons. C. MS showing YFP and NeuN. Note that the majority of YFP was seen in the MS with limited staining of the vertical and horizontal limb of the diagonal band of Broca. 20× magnification. D. Cell counts of the MS (a,c) and CA1 region of the hippocampus (b,d) using DAPI (a,b) or NeuN (c,d). No group differences were seen with either stain or region imaged.

In the CTL group, differences were noted between the learners and non-learners in total (F = 5.234. *p* = 0.0019), theta (F = 12.62, p < 0.0001), slow gamma (F = 5.326, *p* = 0.0062) and med. Gamma coherence (F = 2.113, *p* = 0.0287) (Supplementary Fig. 8C). With PLV group differences were found only in the theta band (F = 3.704, *p* = 0.0139) (Fig. 8D). In CTL rats, learners had higher coherences and PLV values, particularly in the theta band, than the non-learners, regardless of whether they received BL or YL.

This analysis demonstrates a relationship between EEG measures of connectivity and performance in the active avoidance task with higher coherences and PLV associated with improved performance, supporting prior work in our laboratory (Niedecker et al., 2021). In addition, at multiple bands, coherence and PLV values were higher in the ELS-BL learners than the ELS-YL learners.

### 3.4. Histology

Histology was available in almost all the animals: ELS-BL (*n* = 10), ELS-YL (*n* = 11), CTL-BL (n = 10) and CTL-YL (*n* = 9). In three rats, tissue was not available due to technical issues related to perfusion or stain. Other than for electrode tracks, no animals had any gross morphological injury to either the MS or hippocampus. Staining of the hippocampus showed septal axons expressing ChR2-YFP (green) projecting through the stratum oriens to the pyramidal cell layer in all animals (Fig. 8A,B). The YFP-stained neurons were consistently in the medial septum with limited involvement of the vertical and horizontal limbs of the diagonal band of Broca (Fig. 8C). None of the rats had any destructive lesions other than could be accounted for by electrode removal. While electrode tracks were not seen in all rats, when present, the electrodes were consistently in the MS and CA1 region of the hippocampus. NeuN and DAPI-labeled cells were counted in the hippocampus and MS to assess whether BL or YL caused neuron or total-cell loss, respectively. One-way ANOVA showed no differences in density in either DAPI- or NeuN-labeled cells among the four groups in either the MS or CA1 region (Fig. 8D). In summary, the histology demonstrated that the injection of the viral vector into the MS was successful and that optogenetic stimulation did not result in cell loss in the MS or CA1 region.

## 4. Discussion

The goal of this study was to determine whether modulation of dorsal hippocampal oscillations during the critical period of spatial cognitive development can reverse or reduce ELS-induced spatial cognitive deficits and dysrhythmia. Specifically, we investigated whether 7 Hz optogenetic stimulation of the MS would modulate hippocampal theta in rat pups with a history of ELS and if such stimulation had long-term effects of spatial cognition and hippocampal oscillations. Our findings show that stimulation of the MS is a reliable and robust method to regulate hippocampal oscillations in rat pups with and without a history of ELS and that regulation of hippocampal theta during the critical period of spatial cognition improves spatial cognition and oscillatory properties of hippocampal rhythms following ELS.

### 4.1. Optogenetic stimulation of the medial septum in rat pups results in robust regulation of dorsal hippocampus theta in both CTL and ELS rats

A prior study from our laboratory showed that optogenetic stimulation of the MS in P21–25 rats resulted in clear frequency-matched EEGs in CA1 of the hippocampus (Kloc et al., 2020). We replicated and expanded those findings showing that MS optogenetic in P21–25 rats resulted in precise regulation of hippocampal oscillations with frequency-matched EEGs at all theta frequencies in both CTL and ELS rats. There was a high coherence between the MS and hippocampus at all frequencies studied.

When compared to EEG epochs immediately before stimulation, optogenetic stimulation at all theta frequencies between 5 and 12 Hz resulted in increases in intra-hippocampal relative theta power, absolute total and theta power and reductions in coherence and PLV at most theta frequencies. No differences in MS regulation of hippocampal theta were noted in the ELS and CTL groups. As expected, stimulation with YL had no effect on any of the EEG parameters studied.

These findings indicate that during the critical period of spatial memory, MS optogenetic stimulation is a robust and reliable means to modulate hippocampal rhythms in young rats, both with and without a history of ELS.

### 4.2. 7 Hz optogenetic stimulation of the MS during the critical period improves performance in active avoidance in ELS rats

Optogenetic stimulation of the MS with BL resulted in improved performance in the ELS group, as measured by a fewer number of entrances into the shock zone and number of shocks in the active avoidance task. The ELS-YL group were most impaired, with more entrances and shocks than the other three groups. While the 7 Hz BL stimulation resulted in fewer entrances into the shock zone in the CTL-BL group than the CTL-YL group, there were no differences in number of shocks. The 7 Hz BL stimulation paradigm therefore benefited the rat pups undergoing ELS, resulting in behavior like the CTL group. The beneficial effects of optogenetic BL stimulation in CTL pups, if any, is less clear.

In prior studies using active avoidance, we found the CTL rats performed better than seen in this study (Patterson et al., 2017; Kloc et al., 2020; Niedecker et al., 2021; Kloc et al., 2022; Kloc et al., 2021). Control littermates were maternally deprived for equivalent amounts of time as rats undergoing ELS. Maternal deprivation in developing pups can result in cognitive impairment when assessed at an older age (Stoneham et al., 2021; Menezes et al., 2020; Janetsian-Fritz et al., 2018; Yang et al., 2017; Thomas et al., 2016). Maternal deprivation was likely a significant risk factor for spatial cognitive deficits in this study. However, without a CTL group that did not undergo either maternal deprivation or ELS, it is not possible to attribute the poor performance in the CTL group to maternal deprivation. Regardless, the BL stimulation did appear to ameliorate the additional effects of ELS on spatial cognition in rats already vulnerable to negative outcomes because of maternal deprivation.

It is possible that locomotor hyperactivity could contribute to the deficits in the active avoidance task. While locomotor activity was not directly measured, we therefore cannot rule out an effect of hyperactivity on spatial cognition as measured in active avoidance.

### 4.3. 7 Hz optogenetic stimulation of the MS during the critical period modifies EEG in ELS rats

In addition to changing performance in active avoidance in rats following ELS, the 7 Hz stimulations resulted in changes in the post-active avoidance EEG. A higher theta frequency, increase in absolute total and theta power and theta coherence and PLV were seen in ELS rats receiving BL versus YL. While the BL increased absolute total and theta power in the CTL group, the BL did not alter coherences or PLV. Since coherence and PLV are indicators of connectivity, these findings suggest that BL following ELS increases intra-hippocampal functional connectivity. In CTL rats, BL had no discernible effects on functional connectivity.

To determine if there is a relationship between coherence and PLV and spatial cognition, we evaluated the EEGs in the rats classified as learners and non-learners. In general, learners had higher functional measure of connectivity than non-learners and ELS-BL learners higher coherences and PLV than ELS-YL learners. This data suggests that BL following ELS improves cognition through enhanced connectivity as measured by coherence and PLV. However, the relationship between EEG properties and performance in the active avoidance task is correlative and does not prove causation. With only two non-learners in the ELS-BL group, interpretation of the data needs to be done with caution. Also, MS stimulation may have beneficial effects that extend beyond enhancing functional connectivity in the hippocampus. It is known that MS project fibers to multiple brain regions, including the prefrontal cortex. The role of MS stimulation on other brain regions involved in spatial cognition should be investigated.

Of note, the EEGs in this study were obtained in the inactive, but not immobile state. It is possible that obtaining EEG when the animal was actively engaged in the task may have provided additional information since as discussed below, coherence is a dynamic measure that is influenced by behavior.

### 4.4. Relationship between hippocampal oscillations and cognition

There is a clear relationship between coordinated dynamic neural activity within and between neural networks underlying learning and memory (Barry et al., 2016a; Fenton, 2015). Precise coordination of neuronal firing occurring with respect to theta rhythms within the developing hippocampus is necessary for normal spatial cognition (Mizuseki et al., 2009; Douchamps et al., 2013; Siegle and Wilson, 2014; Robbe and Buzsaki, 2009). The dynamic phase relationships of synaptic currents, as well as the timing of action potentials during theta rhythm, are critical in both the encoding and retrieval of memory (Barry and Holmes, 2016). These key features of memory are coordinated by theta phase, which temporally organizes the transfer of neural information within the hippocampus and between the hippocampus and neocortex (Siegle and Wilson, 2014; Hasselmo, 2005). ELS are known to disrupt the normal development of hippocampal oscillations and may account for the cognitive deficits seen following seizures (Holmes et al., 2015; Barry et al., 2015; Patterson et al., 2017; Barry et al., 2020; Barry et al., 2016a; Barry and Holmes, 2016). In this study, animals that demonstrated spatial cognitive deficits following ELS had low theta power, coherences and PLV, indicative of hippocampal dysfunction with deficits in functional neural connectivity. Our finding that 7 Hz MS stimulation following ELS increased relative and absolute theta power, coherences and PLV concomitant with improved spatial cognition suggest that the spatial cognitive deficits are related to ELS-induced dysrhythmia, A further indication that spatial cognitive performance was related to theta properties was our observation that in the ELS-BL group, learners had higher relative theta power and increased coherences and PLV across multiple frequency bands than the non-learners.

Our findings indicate that oscillatory activity is malleable and can be altered with augmentation of hippocampal theta during the critical period. While this is the first study to show that MS optogenetic stimulation in rat pups can result in long-term improvement in theta dynamics, there is data indicating that oscillatory activity is plastic and can be permanently modified with training. (Kleen et al., 2011). Similarly, environment enrichment has been shown to enhance gamma oscillations in the hippocampus (Shinohara et al., 2013), restore theta power following lithium-pilocarpine induced status epilepticus (Vrinda et al., 2017) and normalize hippocampal action potential timing in relationship to theta in an animal model of cortical malformation (Hernan et al., 2018).

The mechanism by which MS optogenetic stimulation during the critical period modifies EEG and enhances spatial cognition was not studied here. In studies of environmental enrichment, genes related to memory formation, synaptic plasticity, and protein synthesis were upregulated in multiple brain regions, including the hippocampus (Hullinger et al., 2015; Lichti et al., 2014; Novkovic et al., 2015; Sampedro-Piquero et al., 2014). Whether MS stimulation has a similar effect as environmental enrichment is not known.

### 4.5. Clinical implications

There is increasing evidence that frequent seizures occurring during early childhood result in both impaired cognition and EEG dysrhythmias. In infants and children with epilepsy, increased slow activity in the delta band and reduced theta and alpha were associated with worse cognitive outcome than children with faster activity on the EEG (Koop et al., 2005; Kulandaivel and Holmes, 2011). In patients with Dravet syndrome, a severe epileptic encephalopathy, the best indicator of cognitive function was the background EEG. Whereas number of seizures and episodes of status epilepticus were not related to outcome, abnormalities of EEG oscillations were correlated with cognitive outcome (Akiyama et al., 2010). Modulating hippocampal oscillations following ELS may provide a novel therapeutic intervention into preventing or reversing subsequent cognitive dysfunction. While doing MS optogenetic stimulation in children with epilepsy is futuristic, our study provides the framework for further exploration into disease modification through adjusting hippocampal oscillations.

However, there are important questions that need answering. It is not known whether the stimulation pattern we used is ideal for improving or reversing cognitive dysfunction following ELS. We reasoned that using a 7 Hz stimulation pattern would be similar to the theta pattern in normal rats at this age (Holmes et al., 2015). The duration of stimulation was partly determined by prior work in our laboratory where Kloc et al. (Kloc et al., 2020) used a random pattern of stimulation frequencies to disrupt hippocampal theta rhythms for either 1Hr or 5 h a day from P21–25 and found this resulted in long-term cognitive impairment. Whether a different frequency or longer or shorter period of stimulation would be more beneficial is unknown. It is known that during learning theta frequency is critical. Finally, it is important to repeat this study using additional behavioral tasks, particularly one not using an aversive stimulus.

## 5. Conclusions

Here we show that MS optogenetic stimulation is a powerful tool in modifying intrinsic hippocampal rhythms in both ELS and CTL animals. MS stimulations deliver clear frequency-matched theta with high power in CA1 of the hippocampus. Intermittent 7 Hz stimulation for several hours per day during the critical period following ELS resulted in modification of subsequent EEGs with high relative and absolute theta power and enhanced coherence and PLV. These changes in the EEG paralleled improved performance in spatial cognition. Furthermore, MS optogenetic stimulation did not result in cell loss in either the ELS or CTL animals. Together, this study provides proof of principle that modifying hippocampal oscillations following ELS can alter dynamic signaling and improve spatial cognition.

## Supplementary Material

Supplemental Table 2

Supplemental Figure 1A

Supplemental Figure 1B

Supplemental Figure 2

Supplemental Figure 3

Supplemental Figure 4

Supplemental Figure 5

Supplemental Figure 7

Supplemental Figure 6

Supplemental Figure 8

Supplemental Table 1

## Data Availability

Data will be made available on request.
