# Supplementary material for: Optogenetic modulation of hippocampal oscillations ameliorates spatial cognition and hippocampal dysrhythmia following early-life seizures
Source: Neurobiol Dis. Author manuscript; Available in PMC 2023 Jul 12. (PMC10338061; doi:10.1016/j.nbd.2023.106021)
Supplement: Supplemental Table 2 [file NIHMS1876324-supplement-Supplemental_Table_2.docx]

| *CTL-BL* | | |  | *ELS-BL* | | |
| --- | --- | --- | --- | --- | --- | --- |
| *Rat Number* | *Electrode Pairs Used* | *Electrode Pairs Discarded* |  | *Rat Number* | *Electrode Pairs Used* | *Electrode Pairs Discarded* |
| CTL1-BL | 9 | 3 |  | ELS11-BL | 10 | 2 |
| CTL2-BL | 5 | 7 |  | ELS12-BL | 8 | 4 |
| CLTL3-BL | 10 | 2 |  | ELS13-BL | 9 | 3 |
| CTL4-BL | 8 | 3 |  | ELS14-Bl | 10 | 2 |
| CLT5-BL | 8 | 3 |  | ELS15-BL | 10 | 2 |
| CTL6-BL | 10 | 2 |  | ELS16-BL | 10 | 2 |
| CLT7-BL | 10 | 2 |  | ELS17-BL | 10 | 2 |
| CTL8-BL | 8 | 4 |  | ELS18-BL | 8 | 4 |
| CTL9-BL | 10 | 2 |  | ELS19-BL | 9 | 3 |
| CLT10-BL | 8 | 4 |  | ELS20-BL | 7 | 5 |
|  |  |  |  | ELS21-BL | 8 | 3 |
| Mean | 8.6±0.50 | 3.2±0.49 |  | | 9.00±0.33 | 2.9±0.31 |
| *CTL-YL* | | |  | *ELS-YL* | | |
| *Rat Number* | *Electrode Pairs Used* | *Electrode Pairs Discarded* |  | *Rat Number* | *Electrode Pairs Used* | *Electrode Pairs Discarded* |
| CTL22-YL | 10 | 2 |  | ELS32-YL | 10 | 2 |
| CTL23-YL | 10 | 2 |  | ELS33-YL | 8 | 4 |
| CTL24-YL | 6 | 6 |  | ELS34-YL | 10 | 2 |
| CTL25-YL | 9 | 3 |  | ELS35-YL | 10 | 2 |
| CTL26-YL | 10 | 2 |  | ELS36-YL | 10 | 2 |
| CTL27-YL | 8 | 4 |  | ELS37-YL | 8 | 4 |
| CTL28-YL | 8 | 4 |  | ELS38-YL | 8 | 4 |
| CTL29-YL | 8 | 4 |  | ELS39-YL | 8 | 4 |
| CTL30-YL | 8 | 4 |  | ELS40-YL | 8 | 4 |
| CTL31-YL | 9 | 3 |  | ELS41-YL | 10 | 2 |
|  |  |  |  | ELS42-YL | 10 | 2 |
| Mean | 8.6±0.40 | 3.4±0.40 |  | | 9.1±0.34 | 2.9±0.31 |

Suppl. Table 2
