# Supplementary material for: Optogenetic modulation of hippocampal oscillations ameliorates spatial cognition and hippocampal dysrhythmia following early-life seizures
Source: Neurobiol Dis. Author manuscript; Available in PMC 2023 Jul 12. (PMC10338061; doi:10.1016/j.nbd.2023.106021)
Supplement: Supplemental Figure 1A [file NIHMS1876324-supplement-Supplemental_Figure_1A.pptx]

## Slide 1
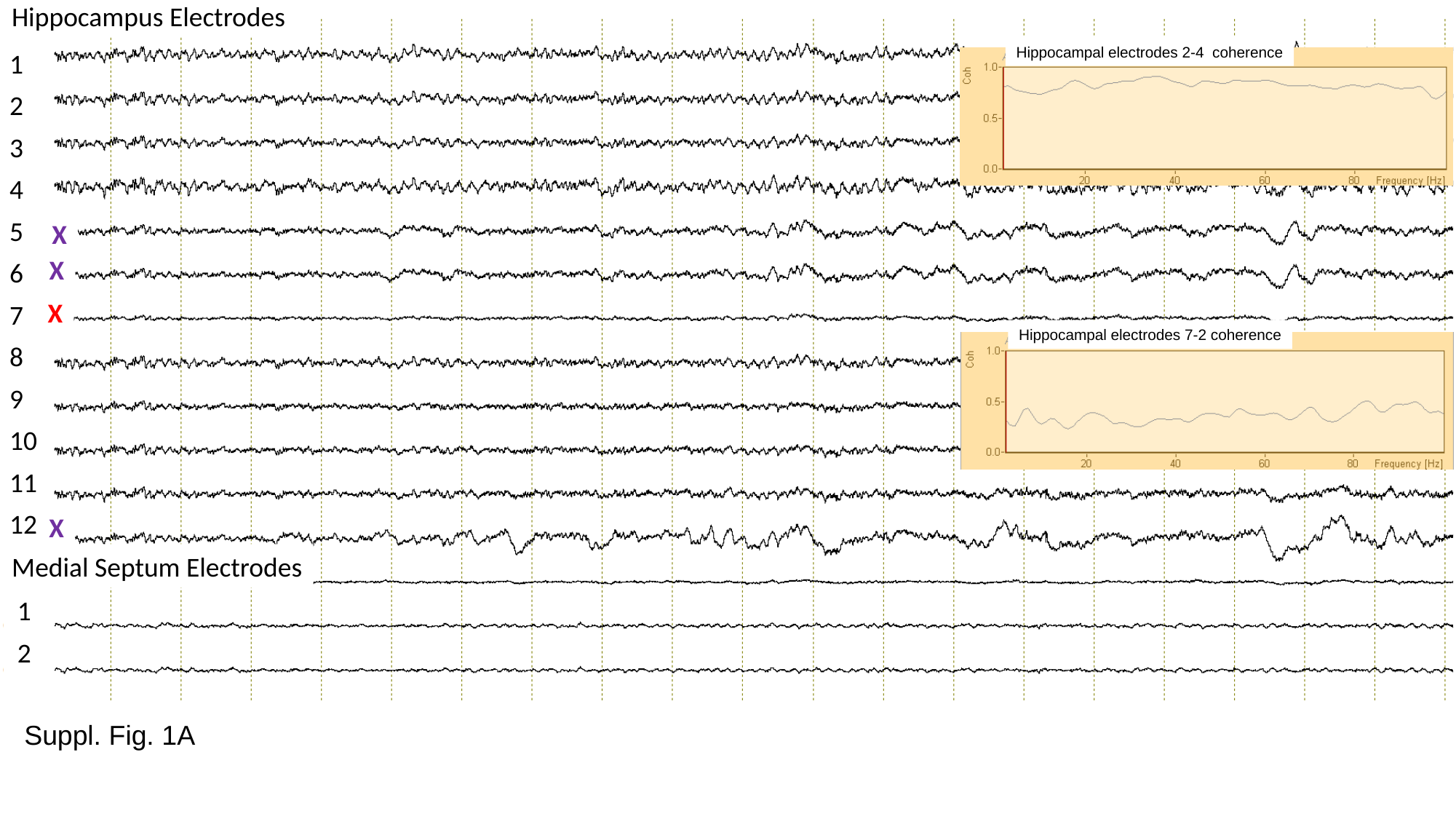

Hippocampus Electrodes
Hippocampal electrodes 2-4 coherence
1
2
3
4
5
6
7
8
9
10
11
12
X
X
X
Hippocampal electrodes 7-2 coherence
X
Medial Septum Electrodes
1
2
1
2
Suppl. Fig. 1A
