# Supplementary material for: Optogenetic modulation of hippocampal oscillations ameliorates spatial cognition and hippocampal dysrhythmia following early-life seizures
Source: Neurobiol Dis. Author manuscript; Available in PMC 2023 Jul 12. (PMC10338061; doi:10.1016/j.nbd.2023.106021)
Supplement: Supplemental Figure 1B [file NIHMS1876324-supplement-Supplemental_Figure_1B.pptx]

## Slide 1
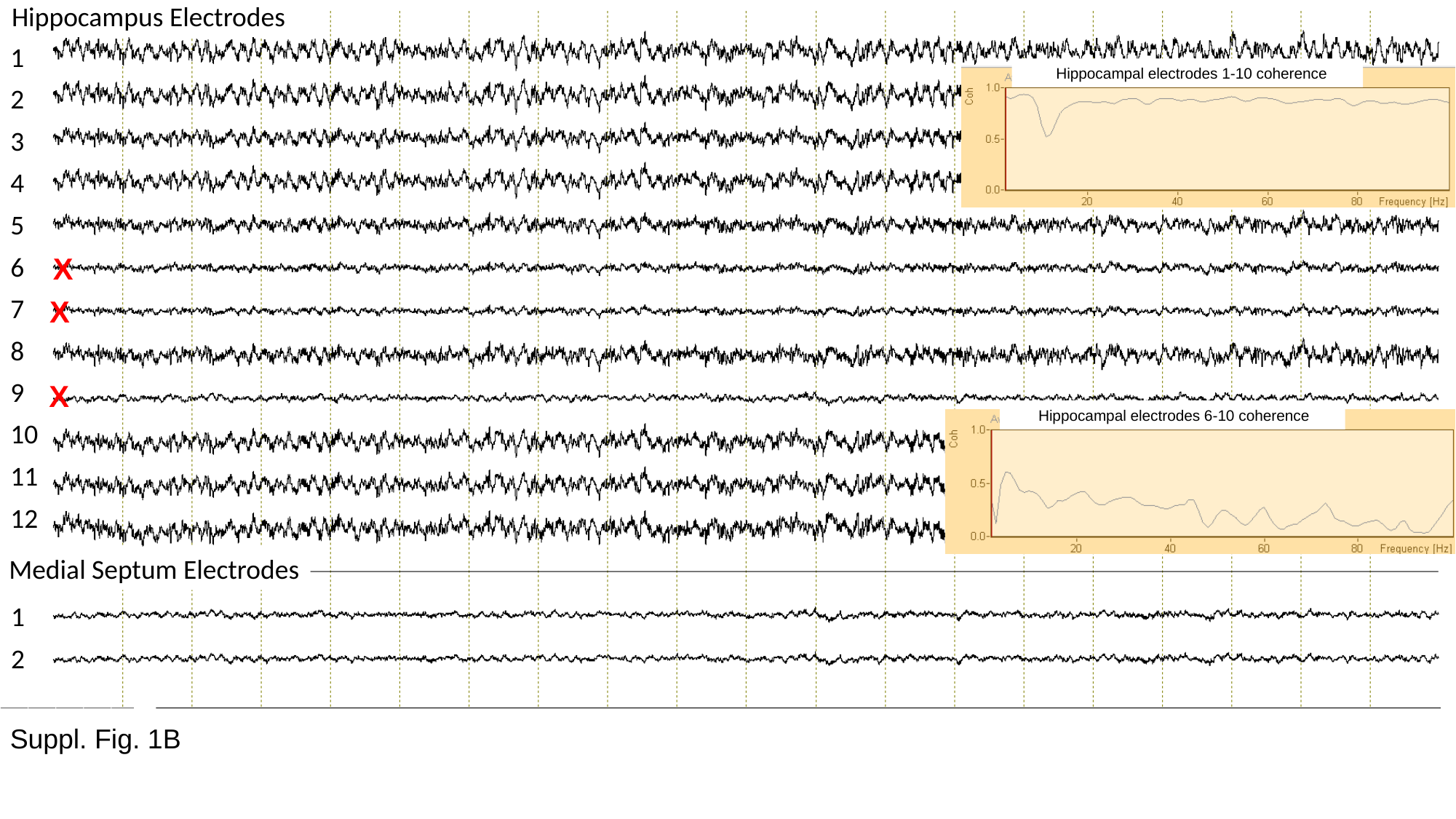

Hippocampus Electrodes
1
2
3
4
5
6
7
8
9
10
11
12
1
2
3
4
5
6
7
8
9
10
11
12
 Hippocampal electrodes 1-10 coherence
X
X
X
 Hippocampal electrodes 6-10 coherence
Medial Septum Electrodes
1
2
Suppl. Fig. 1B
