# Supplementary figures and images for: Optogenetic modulation of hippocampal oscillations ameliorates spatial cognition and hippocampal dysrhythmia following early-life seizures
Source: Neurobiol Dis. Author manuscript; Available in PMC 2023 Jul 12. (PMC10338061; doi:10.1016/j.nbd.2023.106021)

## Slide 1
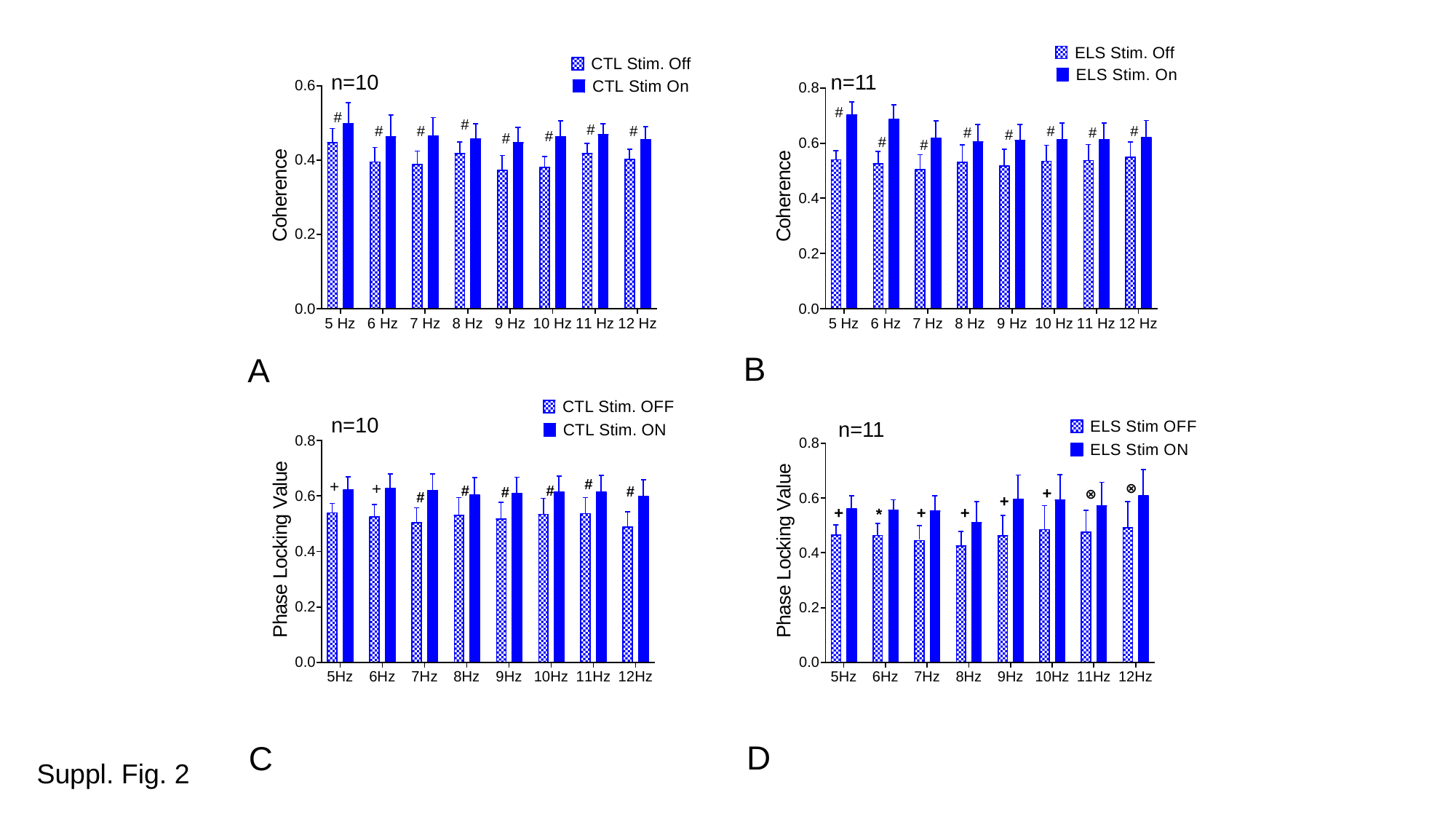

n=11
n=10
n=10
n=11
Suppl. Fig. 2

Supplement: Supplemental Figure 2 [file NIHMS1876324-supplement-Supplemental_Figure_2.pptx]

## Slide 1
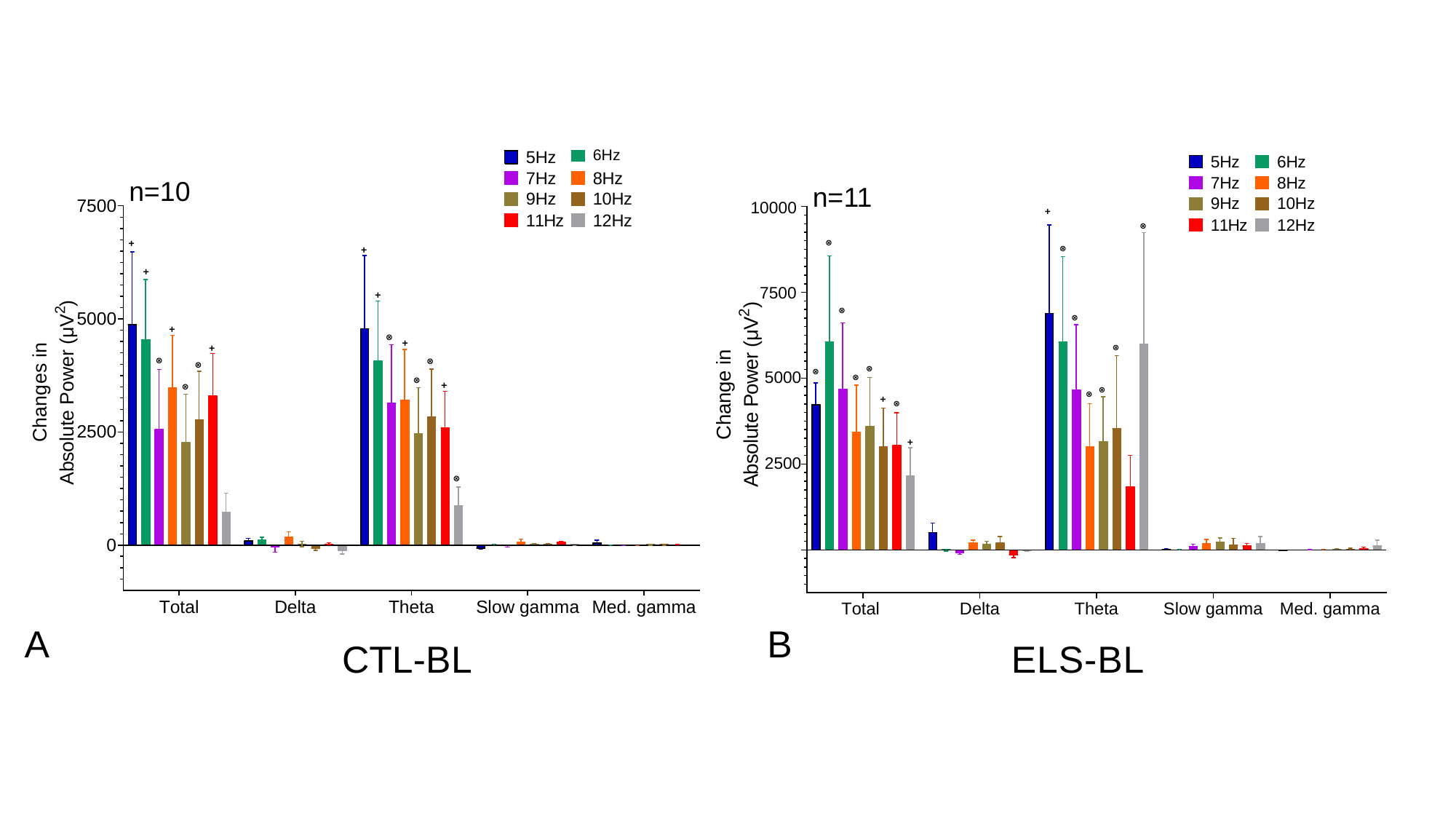

n=10
n=11

Supplement: Supplemental Figure 3 [file NIHMS1876324-supplement-Supplemental_Figure_3.pptx]

## Slide 1
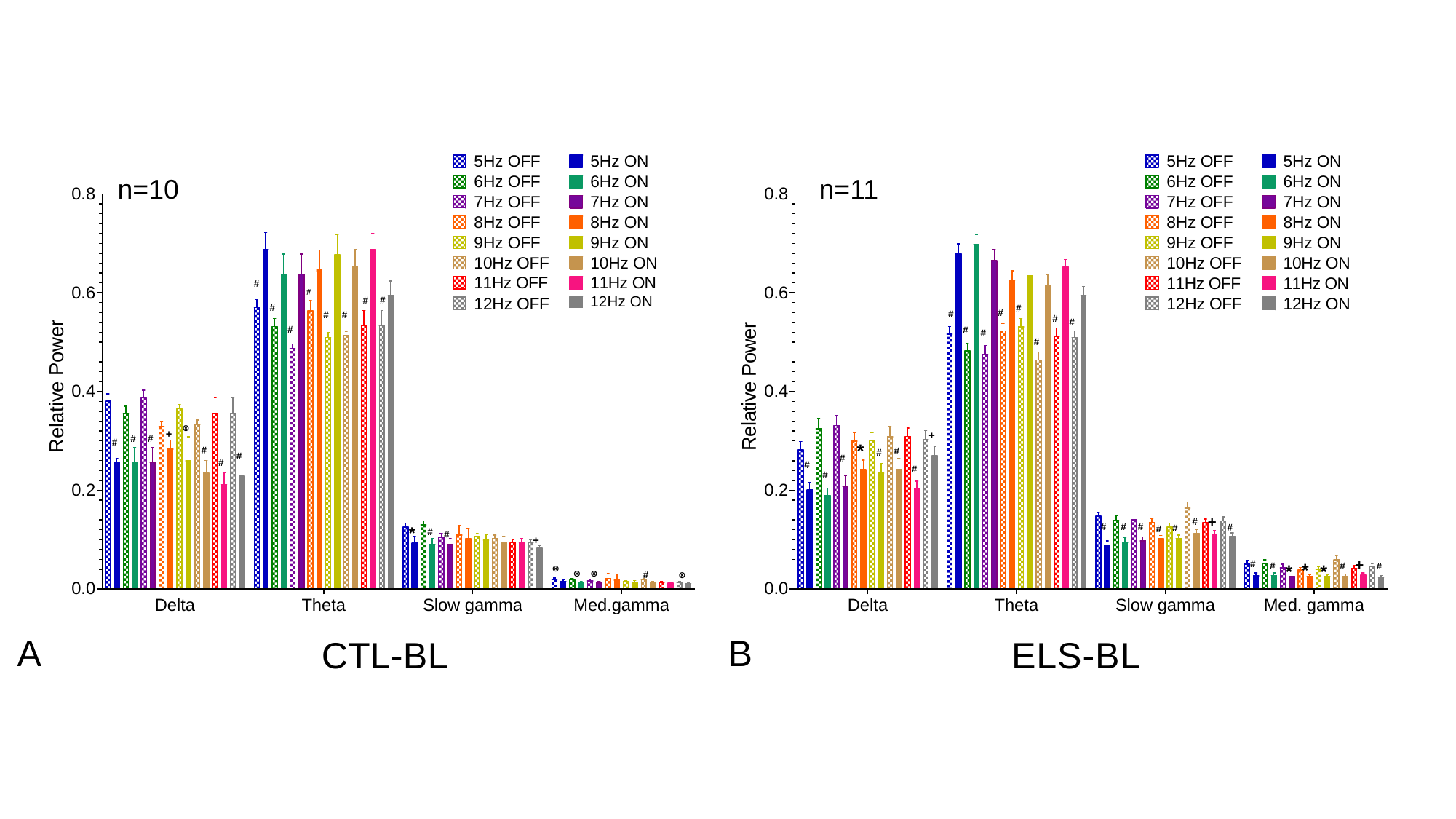

n=10
n=11

Supplement: Supplemental Figure 4 [file NIHMS1876324-supplement-Supplemental_Figure_4.pptx]

## Slide 1
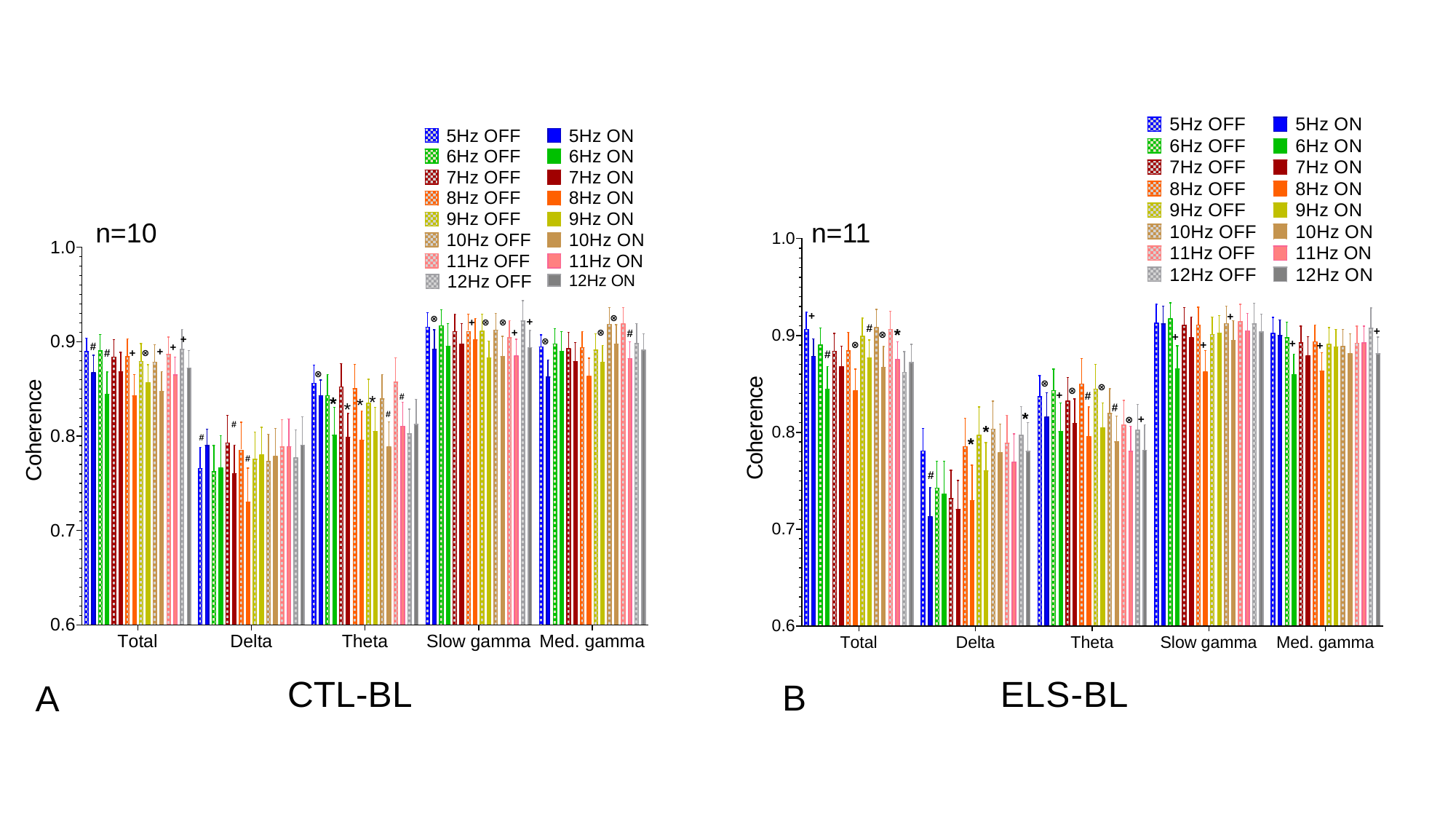

n=10
n=11

Supplement: Supplemental Figure 5 [file NIHMS1876324-supplement-Supplemental_Figure_5.pptx]

## Slide 1
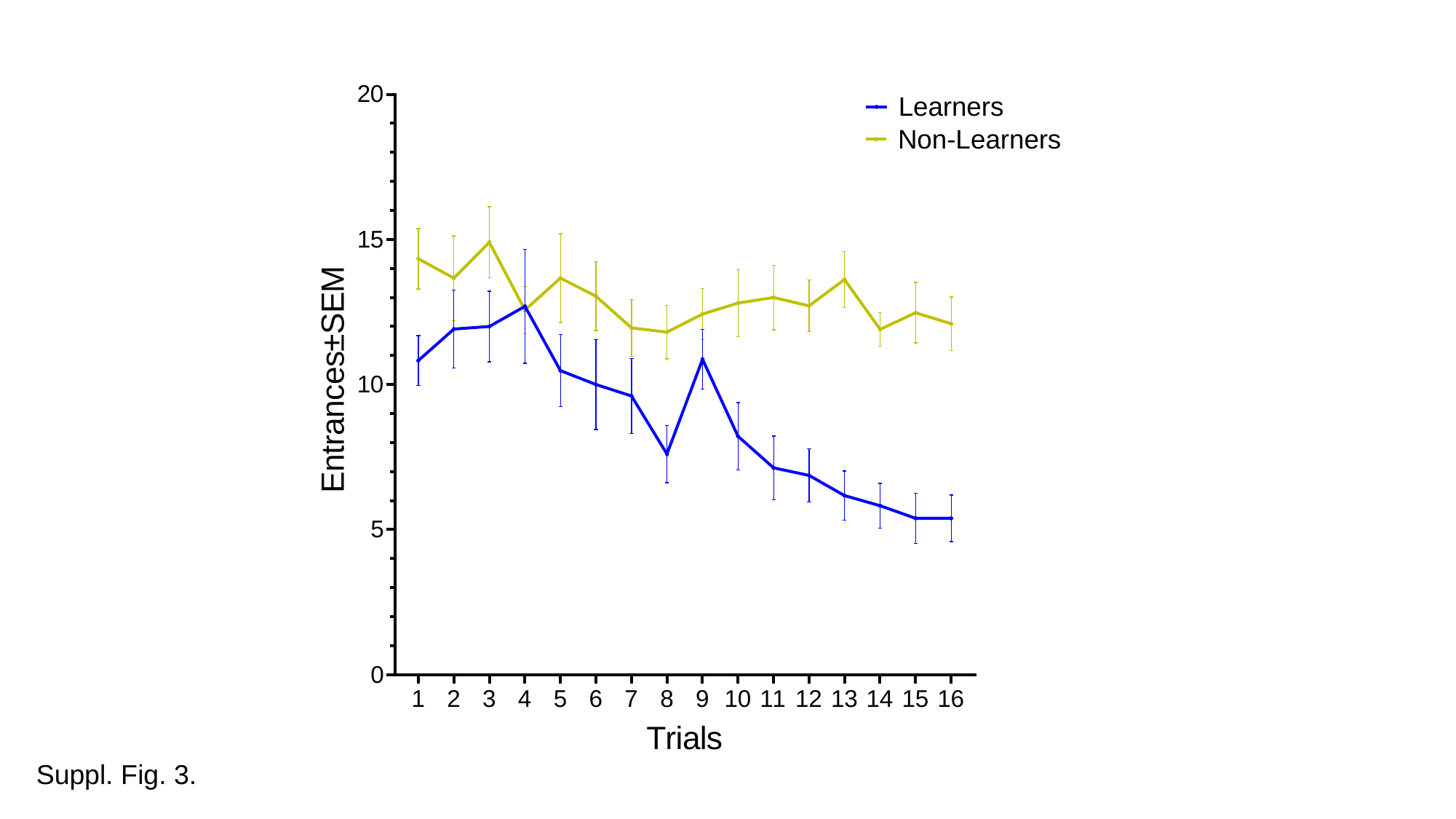

Suppl. Fig. 3.

Supplement: Supplemental Figure 7 [file NIHMS1876324-supplement-Supplemental_Figure_7.pptx]

## Slide 1
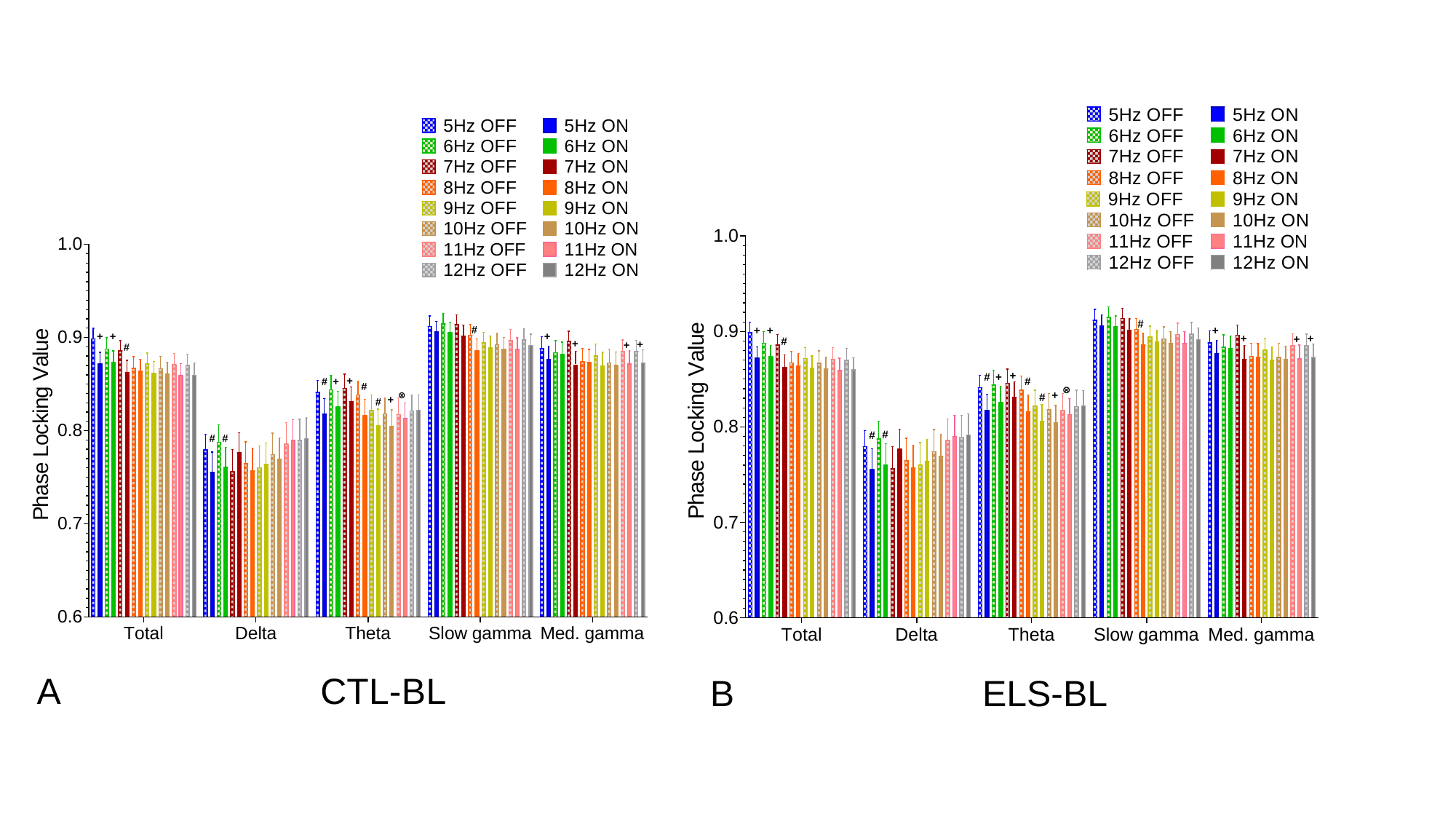

ELS-BL
B

Supplement: Supplemental Figure 6 [file NIHMS1876324-supplement-Supplemental_Figure_6.pptx]

## Slide 1
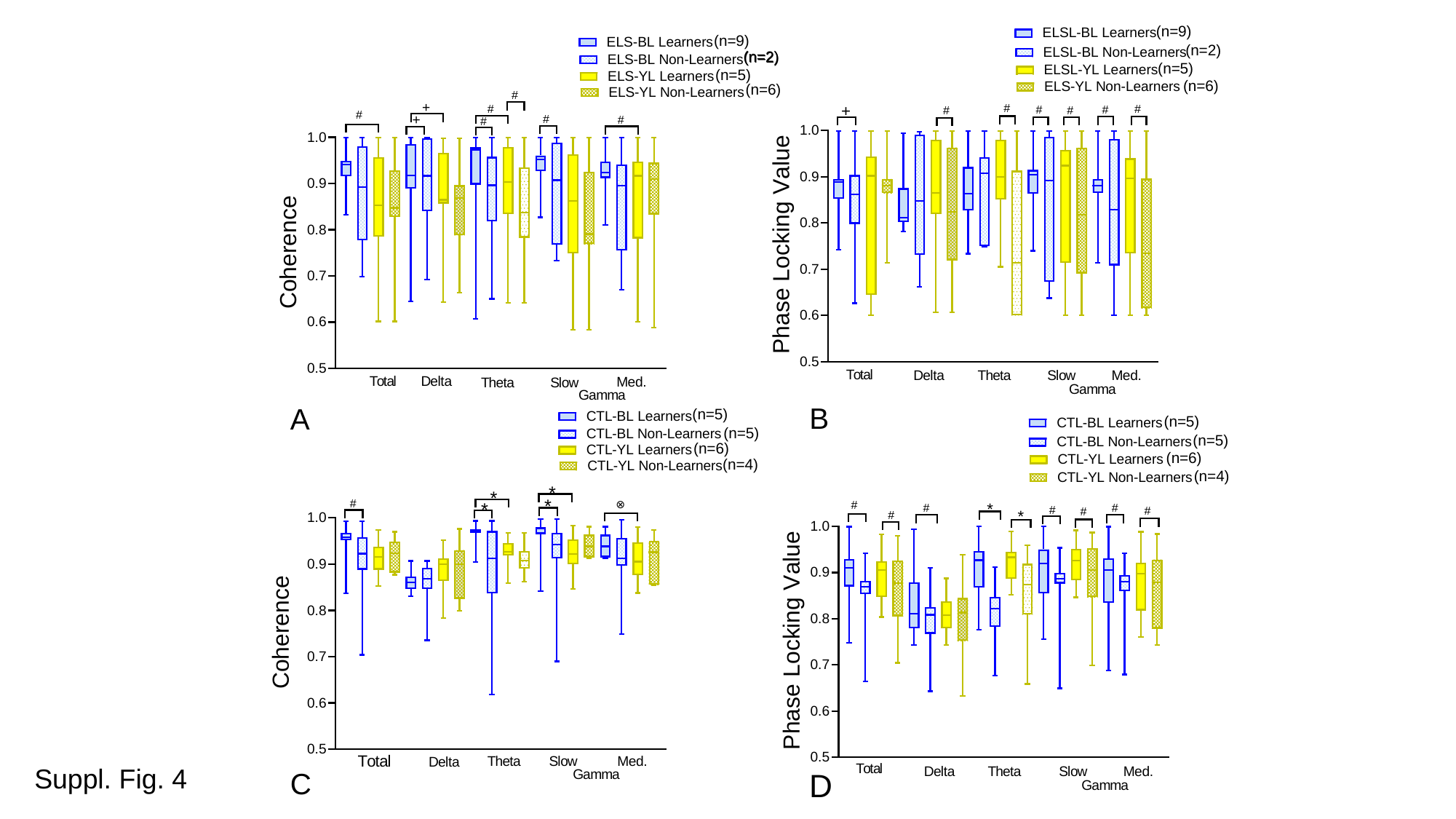

(n=9)
(n=9)
(n=2)
(n=2)
(n=2)
(n=5)
(n=5)
(n=6)
(n=6)
(n=5)
(n=5)
(n=5)
(n=5)
(n=6)
(n=6)
(n=4)
(n=4)
Suppl. Fig. 4

Supplement: Supplemental Figure 8 [file NIHMS1876324-supplement-Supplemental_Figure_8.pptx]
