# Supplementary material for: Optogenetic modulation of hippocampal oscillations ameliorates spatial cognition and hippocampal dysrhythmia following early-life seizures
Source: Neurobiol Dis. Author manuscript; Available in PMC 2023 Jul 12. (PMC10338061; doi:10.1016/j.nbd.2023.106021)
Supplement: Supplemental Table 1 [file NIHMS1876324-supplement-Supplemental_Table_1.docx]

| *CTL-BL* | | | *ELS-BL* | | |
| --- | --- | --- | --- | --- | --- |
| *Rat Number* | *Electrode Pairs Used* | *Electrode Pairs Discarded* | *Rat Number* | *Electrode Pairs Used* | *Electrode Pairs Discarded* |
| CTL1-BL | 10 | 2 | ELS11-BL | 10 | 2 |
| CTL2-BL | 5 | 7 | ELS12-BL | 8 | 4 |
| CLTL3-BL | 10 | 2 | ELS13-BL | 9 | 3 |
| CTL4-BL | 8 | 3 | ELS14-Bl | 10 | 2 |
| CLT5-BL | 8 | 3 | ELS15-BL | 10 | 2 |
| CTL6-BL | 10 | 2 | ELS16-BL | 10 | 2 |
| CLT7-BL | 10 | 2 | ELS17-BL | 10 | 2 |
| CTL8-BL | 8 | 4 | ELS18-BL | 9 | 3 |
| CTL9-BL | 10 | 2 | ELS19-BL | 9 | 3 |
| CLT10-BL | 8 | 4 | ELS20-BL | 7 | 5 |
|  |  |  | ELS21-BL | 8 | 3 |
| Mean | 8.7±0.52 | 3.1±0.50 |  | 9.09±0.31 | 2.8±0.29 |
| *CTL-BL* | | | *ELS-YL* | | |
| *Rat Number* | *Electrode Pairs Used* | *Electrode Pairs Discarded* | *Rat Number* | *Electrode Pairs Used* | *Electrode Pairs Discarded* |
| CTL22-YL | 10 | 2 | ELS32-YL | 10 | 2 |
| CTL23-YL | 10 | 2 | ELS33-YL | 8 | 4 |
| CTL24-YL | 6 | 6 | ELS34-YL | 10 | 2 |
| CTL25-YL | 9 | 3 | ELS35-YL | 10 | 2 |
| CTL26-YL | 10 | 2 | ELS36-YL | 10 | 2 |
| CTL27-YL | 9 | 3 | ELS37-YL | 9 | 3 |
| CTL28-YL | 8 | 4 | ELS38-YL | 8 | 4 |
| CTL29-YL | 10 | 2 | ELS39-YL | 10 | 2 |
| CTL30-YL | 9 | 3 | ELS40-YL | 8 | 4 |
| CTL31-YL | 9 | 3 | ELS41-YL | 10 | 2 |
|  |  |  | ELS42-YL | 10 | 2 |
| Mean | 9.0±0.39 | 3.0±0.39 |  | 9.36±0.28 | 2.6±0.28 |

Suppl. Table 1
